# Lactylation‐Mediated HER2 Stabilization Promotes Trastuzumab Resistance in Gastric Cancer

**DOI:** 10.1002/advs.77955

**Published:** 2026-10-11

**Authors:** Meng Qian, Tongguan Tian, Zhenxiang Wang, Huihui Sun, Ming Zong, Ying Chen, Jie Xiong, Ye Chen, Zhiqian Hu, Tianming Li, Yanping Xu, Shuchang Xu

**Affiliations:** ^1^ Department of Gastroenterology Tongji Institute of Digestive Disease School of Medicine Tongji Hospital Tongji University Shanghai China; ^2^ Shanghai Key Laboratory of Signaling and Disease Research Frontier Science Center For Stem Cell Research School of Life Sciences and Technology Tongji University Shanghai China; ^3^ School of Life Sciences and Technology Tongji University Shanghai China; ^4^ Department of Gastrointestinal Surgery Tongji Hospital Tongji University School of Medicine Shanghai China

**Keywords:** gastric cancer, human epidermal growth factor receptor 2 (HER2), lactylation, resistance mechanism, trastuzumab

## Abstract

Trastuzumab is the standard first‐line targeted therapy for human epidermal growth factor receptor 2 (HER2)‐positive gastric cancer (GC). Unfortunately, its clinical efficacy is often compromised by the development of drug resistance. The Warburg effect, a hallmark of cancer metabolism, leads to substantial lactate accumulation. Nevertheless, the impact of metabolic reprogramming on the response to HER2‐targeted therapy remains poorly understood. Here, we demonstrate that AARS1‐mediated lactylation of HER2 promotes trastuzumab resistance in GC. Mechanistically, lactylation at lysine 1171 (K1171) promotes USP11‐mediated deubiquitination and stabilization of HER2 and also enhances the interaction between HER2 and EGFR. Clinically, elevated HER2 K1171 lactylation levels are associated with poor prognosis in GC patients. Inhibiting HER2 K1171 lactylation with L‐alanine or stiripentol—a lactate dehydrogenase inhibitor (LDHi) clinically approved for epilepsy—significantly sensitizes tumor cells to trastuzumab. Furthermore, a cell‐penetrating peptide designed to specifically block HER2 K1171 lactylation potently enhances antitumor efficacy in vivo. In summary, our findings identify HER2 lactylation as a critical mechanism underlying trastuzumab resistance and highlight lactate metabolism as a promising therapeutic target for HER2‐positive GC.

## Graphical abstract

Warburg effect‐derived lactate drives HER2 lactylation and trastuzumab resistance. Mechanistically, AARS1‐mediated lactylation stabilizes HER2 by inhibiting USP11‐dependent degradation and concurrently promotes HER2‐EGFR interaction. Importantly, inhibiting HER2 lactylation resensitizes gastric cancer cells to trastuzumab. Furthermore, targeting lactate metabolism may benefit patients with HER2‐low expression.

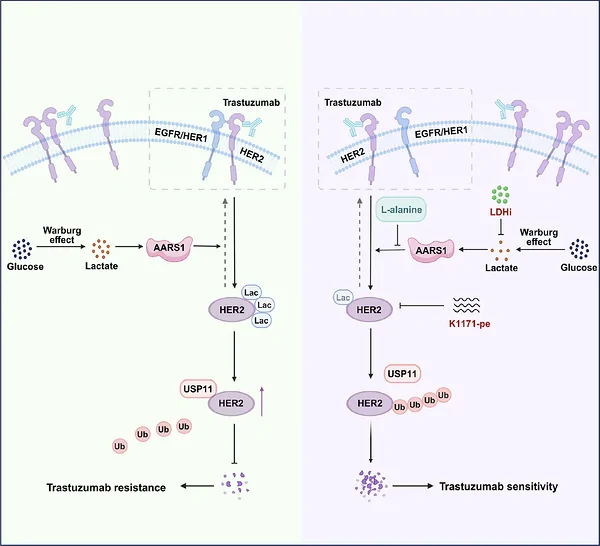

## 1 Introduction

Gastric cancer (GC) is one of the most prevalent malignancies worldwide, ranking fifth in both incidence and mortality. Human epidermal growth factor receptor 2 (HER2) is a well‐established therapeutic target in oncology, with amplification or overexpression observed in approximately 20% of GC cases [1]. Clinical guidelines recommend trastuzumab in combination with chemotherapy as the standard first‐line treatment for HER2‐positive advanced GC [2, 3]. However, the inevitable emergence of trastuzumab resistance presents a significant clinical challenge [4]. Therefore, elucidating the molecular mechanisms underlying trastuzumab resistance and developing effective targeted therapeutic strategies are imperative for improving the prognosis of patients with HER2‐positive GC.

The mechanisms underlying trastuzumab resistance in HER2‐positive GC are multifaceted and remain incompletely elucidated. Accumulating evidence suggests that the inability of current inhibitors to effectively induce HER2 receptor degradation contributes significantly to resistance [5, 6]. Consequently, promoting HER2 degradation may be an effective strategy for overcoming resistance to HER2‐targeted therapies. Acetyltanshinone IIA, a novel small‐molecule compound obtained from Tanshinone IIA, has been shown to significantly reduce HER2 protein levels and thus overcome resistance to HER2‐targeted therapies [7]. Notably, HER2 lacks direct ligand‐binding activity. Instead, overexpressed HER2 activates oncogenic signaling pathways primarily through homodimerization or heterodimerization with other members of the epidermal growth factor receptor (EGFR) family, including EGFR (HER1), HER3, and HER4 [8, 9]. As the preferred dimerization partner for other EGFR family members, HER2 poses a significant challenge to pharmacological inhibition by promoting heterodimerization [10, 11]. Increased HER2/EGFR heterodimerization has been shown to reduce trastuzumab internalization and compromise its antitumor efficacy, a mechanism closely associated with the development of resistance to HER2‐targeted therapies [12].

The Warburg effect is widely recognized as a metabolic hallmark of cancer, leading to substantial lactate accumulation (a glycolytic product) in cancer cells [13]. Growing evidence indicates that lactate plays an important role in tumorigenesis and treatment resistance [14, 15]. Recently, lactylation of lysine residues has been identified as a novel post‐translational modification (PTM) that regulates diverse biological functions by modulating gene expression and protein activity [16, 17]. Previous studies have demonstrated that trastuzumab‐resistant GC cells exhibit heightened glycolytic activity, and that inhibiting glycolysis can significantly enhance trastuzumab efficacy in HER2‐positive GC [18]. However, the role of HER2 lactylation in regulating trastuzumab sensitivity in GC remains unclear.

In this study, we observed elevated lactate levels in tumor tissues from patients with trastuzumab‐resistant GC, and enhanced lactate metabolism was associated with poor prognosis. Mechanistically, lactylation of HER2 enhances its protein stability and promotes HER2‐EGFR binding, thereby contributing to trastuzumab resistance. In addition, we designed a cell‐penetrating peptide that specifically inhibits HER2 lactylation at the K1171 site. This peptide significantly enhanced antitumor efficacy in HER2‐positive GC models. In conclusion, our findings establish HER2 lactylation as a critical mechanism underlying trastuzumab resistance in GC and suggest that targeting HER2 lactylation represents a promising therapeutic strategy to overcome trastuzumab resistance in HER2‐positive GC.

## 2 Results

### 2.1 Lactate Promotes Trastuzumab Resistance and Enhances HER2 Protein Stability

To investigate the metabolic basis of trastuzumab resistance, we performed untargeted metabolomic analyses on postoperative tumor specimens from HER2‐positive GC patients. All enrolled patients received trastuzumab plus chemotherapy as the standard first‐line treatment, and detailed clinical information is summarized in Table S1. Six of the enrolled patients were sensitive to trastuzumab therapy, while six were resistant. Among the 962 identified metabolites, lactate was among the most abundant metabolites in tumors from patients who subsequently developed trastuzumab resistance (Table S2 and Figure 1a). Quantification using a lactate assay kit revealed significantly higher lactate levels in the trastuzumab‐resistant group than in the trastuzumab‐sensitive group (Figure 1b). Furthermore, enzyme‐linked immunosorbent assay (ELISA) analysis of HER2 protein expression in these 12 specimens showed that tissues with high lactate content exhibited significant upregulation of HER2 expression (Figure 1c). Treatment with lactate or sodium lactate (NALA) significantly increased HER2 expression in GC cells, including the AGS and HER2‐positive NCI‐N87 cell lines. Conversely, exposure to the lactate dehydrogenase inhibitors (LDHi) sodium oxamate or stiripentol led to a marked downregulation of HER2 expression (Figure 1d–k). This regulatory effect was also observed in 293T cells (Extended Data Figure S1a–d). Notably, alterations in HER2 protein levels were independent of *HER2* mRNA expression (Extended Data Figure S1e–p). The role of lactate in modulating HER2 protein expression was further confirmed by flow cytometric analysis (Figure 1l,m and Extended Data Figure S1q).

**FIGURE 1:**
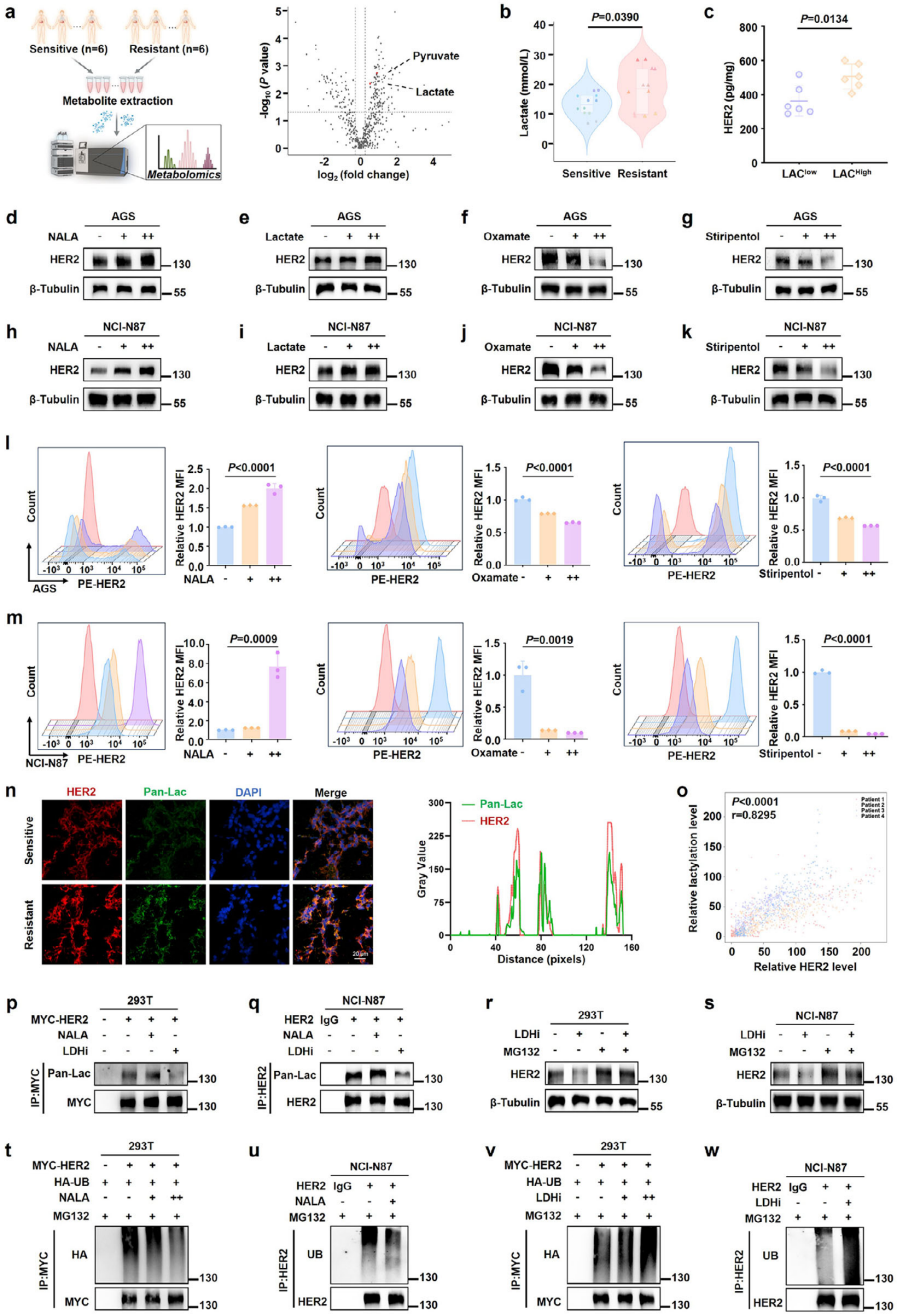
Lactate promotes trastuzumab resistance in gastric cancer by stabilizing HER2 expression. (a) Volcano plot illustrating metabolic differences between gastric cancer tissues from patients who subsequently developed trastuzumab resistance (*n* = 6) and those who remained trastuzumab‐sensitive (*n* = 6), as determined by untargeted metabolomics analysis. All tissues were surgically resected prior to trastuzumab treatment, and sensitivity or resistance to trastuzumab was subsequently determined based on clinical pathology data and patient follow‐up information. Red dots represent metabolites involved in the glycolytic pathway. (b) Lactate levels in gastric cancer specimens measured using a colorimetric assay kit (all samples assayed in duplicate). Each patient sample is shown in a distinct color, and the two technical replicates from the same patient are shown in the same color. (c) HER2 protein levels measured by enzyme‐linked immunosorbent assay (ELISA) in gastric cancer samples (LAC^low^, *n* = 6; LAC^High^, *n* = 6). (d,e) HER2 protein levels in AGS cells following treatment with sodium lactate (NALA, 25 or 50 mm) and lactate (10 or 20 mm). (f,g) HER2 protein levels in AGS cells following treatment with sodium oxamate (10 or 20 mm) and stiripentol (100 or 200 µm). (h,i) HER2 protein levels in NCI‐N87 cells following treatment with NALA (25 or 50 mm) and lactate (10 or 20 mm). (j,k) HER2 protein levels in NCI‐N87 cells following treatment with sodium oxamate (10 or 20 mm) and stiripentol (100 or 200 µm). (l) Flow cytometric analysis of HER2 protein expression in AGS cells treated with NALA or lactate dehydrogenase inhibitors (LDHi) (*n* = 3). (m) Flow cytometric analysis of HER2 protein expression in NCI‐N87 cells treated with NALA or LDHi (*n* = 3). (n) Representative immunofluorescence (IF) staining and co‐localization analysis of pan‐lactylation and HER2 in gastric cancer tissues (scale bars, 20 µm). (o) Correlation between pan‐lactylation and HER2 expression in gastric cancer tissues (Spearman correlation analysis). (p,q) Lactylation levels of exogenous and endogenous HER2 in cells treated with NALA or LDHi. (r,s) HER2 protein levels in 293T and NCI‐N87 cells treated with LDHi and/or MG132 (10 µm). (t,u) Ubiquitination of exogenous and endogenous HER2 following NALA treatment. (v,w) Ubiquitination of exogenous and endogenous HER2 following LDHi treatment. *p*‐values were determined by Student's *t*‐test; ns, not significant. Data are presented as mean ± SD.

Pan‐lactylation levels were significantly elevated in trastuzumab‐resistant tissues compared with trastuzumab‐sensitive tissues (Figure 1n and Extended Data Figure S1r). Meanwhile, a strong positive correlation was observed between HER2 protein expression and global lactylation levels (Figure 1o). In cells pretreated with NALA or LDHi, lactylation of both exogenously introduced and endogenously expressed HER2 was accordingly modulated (Figure 1p,q). To further identify the proteolytic pathway responsible for HER2 degradation, cells were treated with inhibitors targeting two major proteolytic pathways: the 26S proteasome (MG132) and the lysosome (NH4Cl). MG132 treatment resulted in significant HER2 protein accumulation (Extended Data Figure S1s). Moreover, co‐treatment with MG132 completely abolished the LDHi‐induced reduction in HER2 protein levels (Figure 1r,s). Importantly, NALA treatment decreased the ubiquitination of both exogenously and endogenously expressed HER2, whereas LDHi treatment increased it (Figure 1t–w).

### 2.2 AARS1‐Mediated HER2 K1171 Lactylation Enhances Its Protein Stability

To identify the writer responsible for HER2 lactylation, we performed immunoaffinity purification followed by liquid chromatography‐mass spectrometry (LC‐MS/MS) analysis (Table S3). Based on differential enrichment and functional relevance, we screened candidate proteins, including KAT2A and AARS1, and identified AARS1 as the primary writer of HER2 lactylation (Figure 2a,b). Co‐immunoprecipitation (Co‐IP) assays confirmed the interaction between HER2 and AARS1 (Figure 2c,d and Extended Data Figure S2a). Furthermore, an in vitro lactylation assay, using purified recombinant HER2 protein as the substrate, demonstrated that AARS1 directly mediates HER2 lactylation (Figure 2e). Consistent with these findings, immunofluorescence (IF) staining showed strong colocalization of HER2 and AARS1 in both human tissues and GC cells (Figure 2f,g).

**FIGURE 2:**
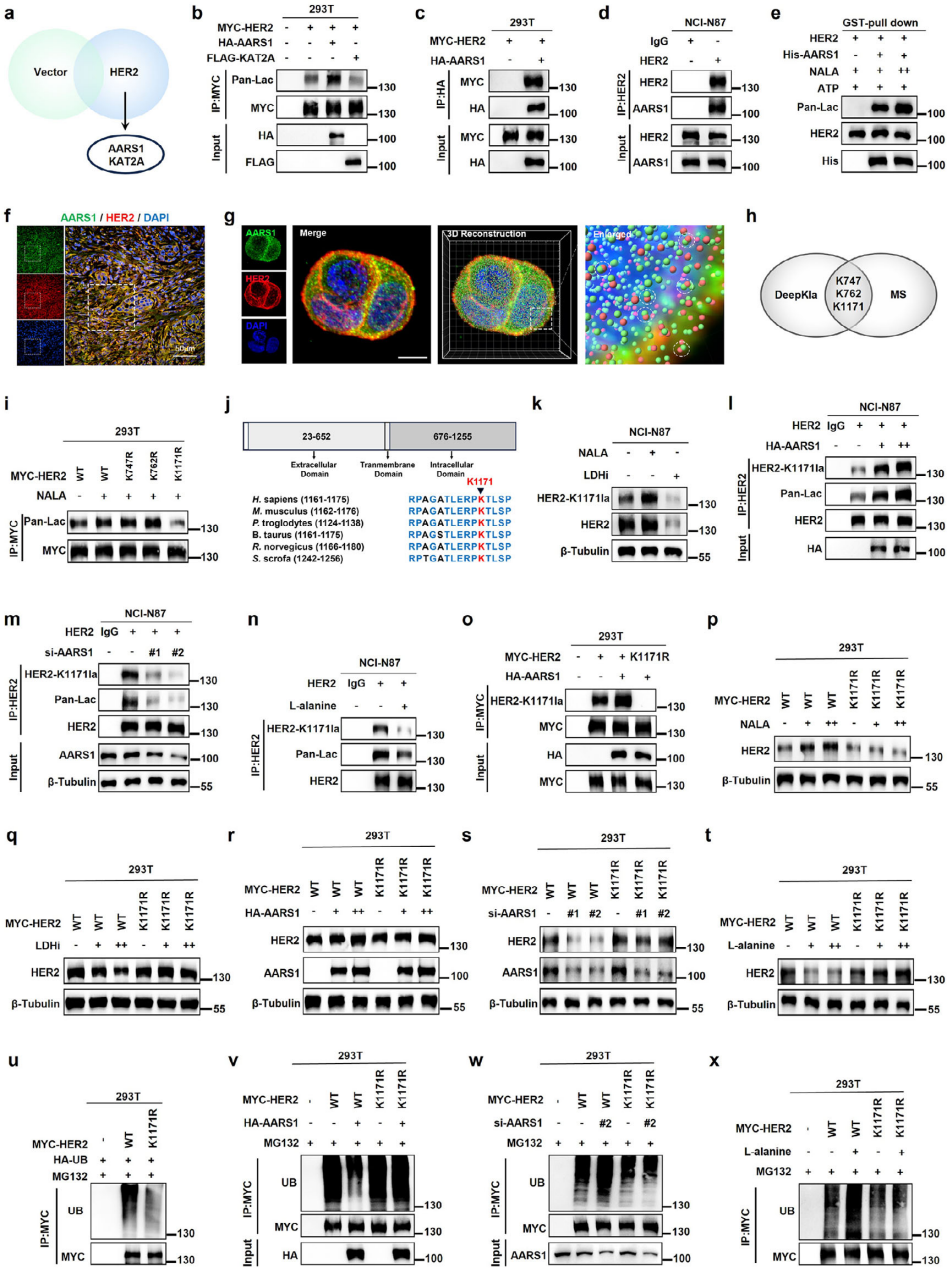
AARS1‐mediated HER2 K1171 lactylation enhances its protein stability. (a) Identification of candidate proteins for HER2 lactylation by liquid chromatography‐mass spectrometry (LC‐MS/MS). (b) Screening for the “writer” of HER2 lactylation using co‐immunoprecipitation (Co‐IP). (c) Exogenous Co‐IP assay confirming the interaction between HER2 and AARS1 in 293T cells. (d) Endogenous Co‐IP assays confirming the interaction in NCI‐N87 cells. (e) In vitro lactylation assay showing that AARS1 directly mediates HER2 lactylation. (f,g) IF staining showing the co‐localization of HER2 and AARS1 in human gastric cancer tissue (scale bars, 50 µm) (f) and NCI‐N87 cells (scale bars, 5 µm) (g). (h) Integrating the lactylation sites identified by LC‐MS/MS with DeepKla predictions. (i) Identification of the lactylation site in HER2. (j) Conservation analysis of the lactylation site in HER2 orthologs across species. (k) Western blot analysis of HER2 lactylation in NCI‐N87 cells treated with NALA or LDHi. (l–n) HER2 K1171 lactylation levels following AARS1 overexpression (l), AARS1 knockdown (m), and L‐alanine treatment (n). (o) Lactylation levels of HER2 wild‐type (WT) and the K1171R mutant following AARS1 overexpression. (p,q) Protein levels of HER2 WT and HER2 K1171R mutant following NALA or LDHi treatment. (r–t) Protein levels of HER2 WT and HER2 K1171R mutant following AARS1 overexpression (r), AARS1 knockdown (s), and L‐alanine treatment (t). (u) Western blot analysis of ubiquitination levels in HER2 and the K1171 mutant. (v–x) Ubiquitination of HER2 WT and HER2 K1171R mutant following AARS1 overexpression (v), AARS1 knockdown (w), and L‐alanine treatment (x).

We next performed LC‐MS/MS analysis to identify potential HER2 lactylation sites. By integrating the nine sites identified by LC‐MS/MS with predictions from DeepKla (http://lin‐group.cn/server/DeepKla/), a computational tool for predicting protein lysine lactylation sites, we identified three candidate HER2 lactylation sites: K747, K762, and K1171 (Figure 2h and Extended Data Figure S2b–d). We further determined that K1171 is the primary lactylation site of HER2 and that this residue is highly conserved across different species (Figure 2i,j). To enable the specific recognition of HER2 K1171 lactylation, a site‐specific antibody (HER2‐K1171la) was generated. The specificity of anti‐HER2‐K1171la was validated by the loss of signal in the HER2 K1171R mutant and by dot blot assay (Extended Data Figure S2e,f). We found that NALA treatment robustly enhanced HER2 K1171 lactylation, whereas LDHi treatment markedly reduced this modification (Figure 2k). Consistently, AARS1 overexpression increased K1171 lactylation on endogenous HER2 in a concentration‐dependent manner, while AARS1 knockdown decreased it (Figure 2l,m). L‐alanine treatment also significantly reduced HER2 K1171 lactylation by competing with lactate for binding to AARS1 (Figure 2n). Notably, AARS1 overexpression markedly increased lactylation of HER2 wild‐type (WT), but not that of HER2 K1171R mutant (Figure 2o).

Furthermore, NALA treatment upregulated the protein expression of HER2 WT but not the K1171R mutant, whereas LDHi treatment downregulated HER2 WT protein expression without affecting the mutant (Figure 2p,q). Similarly, AARS1 overexpression significantly increased HER2 WT protein levels, while AARS1 knockdown or L‐alanine treatment markedly decreased them. However, none of these interventions altered protein levels of the HER2 K1171R mutant (Figure 2r–t). To investigate whether AARS1 directly regulates HER2 protein expression, we examined HER2 mRNA levels in NCI‐N87 cells with AARS1 overexpression or knockdown. Neither perturbation caused significant changes in HER2 mRNA levels (Extended Data Figure S2g,h). Western blot analysis showed that MG132 treatment significantly increased HER2 protein levels. Notably, AARS1 overexpression did not further increase HER2 protein levels in the presence of MG132, compared with MG132 treatment alone (Extended Data Figure S2i). Taken together, these results indicate that AARS1 stabilizes HER2 protein by inhibiting its proteasomal degradation, rather than by promoting protein synthesis.

To further explore the mechanism underlying this post‐transcriptional regulation, we next investigated the role of K1171 lactylation in ubiquitination. Ectopic ubiquitin expression significantly increased the ubiquitination level of HER2 WT but not that of the K1171R mutant (Figure 2u). Consistently, AARS1 overexpression decreased HER2 WT ubiquitination, while AARS1 knockdown or L‐alanine treatment increased it. In contrast, none of these manipulations affected the ubiquitination level of the HER2 K1171R mutant (Figure 2v–x).

### 2.3 K1171 Lactylation of HER2 Promotes Its Interaction With USP11

The deubiquitinase (DUB) inhibitor PR619 substantially reduced HER2 protein levels, and even co‐treatment with NALA failed to restore HER2 expression (Figure 3a,b). Proteomic analysis identified nine DUBs that interact with HER2 (Figure 3c). We next evaluated the effect of knocking out each of the nine DUBs on HER2 stability and found that USP11 depletion markedly reduced HER2 protein expression (Figure 3d,e). Overexpression of USP11 WT, but not its catalytically inactive mutant, substantially increased HER2 protein levels in a concentration‐dependent manner (Figure 3f,g). Consistent with this, USP11 WT markedly attenuated HER2 ubiquitination, whereas its inactive mutant had no effect (Figure 3h,i).

**FIGURE 3:**
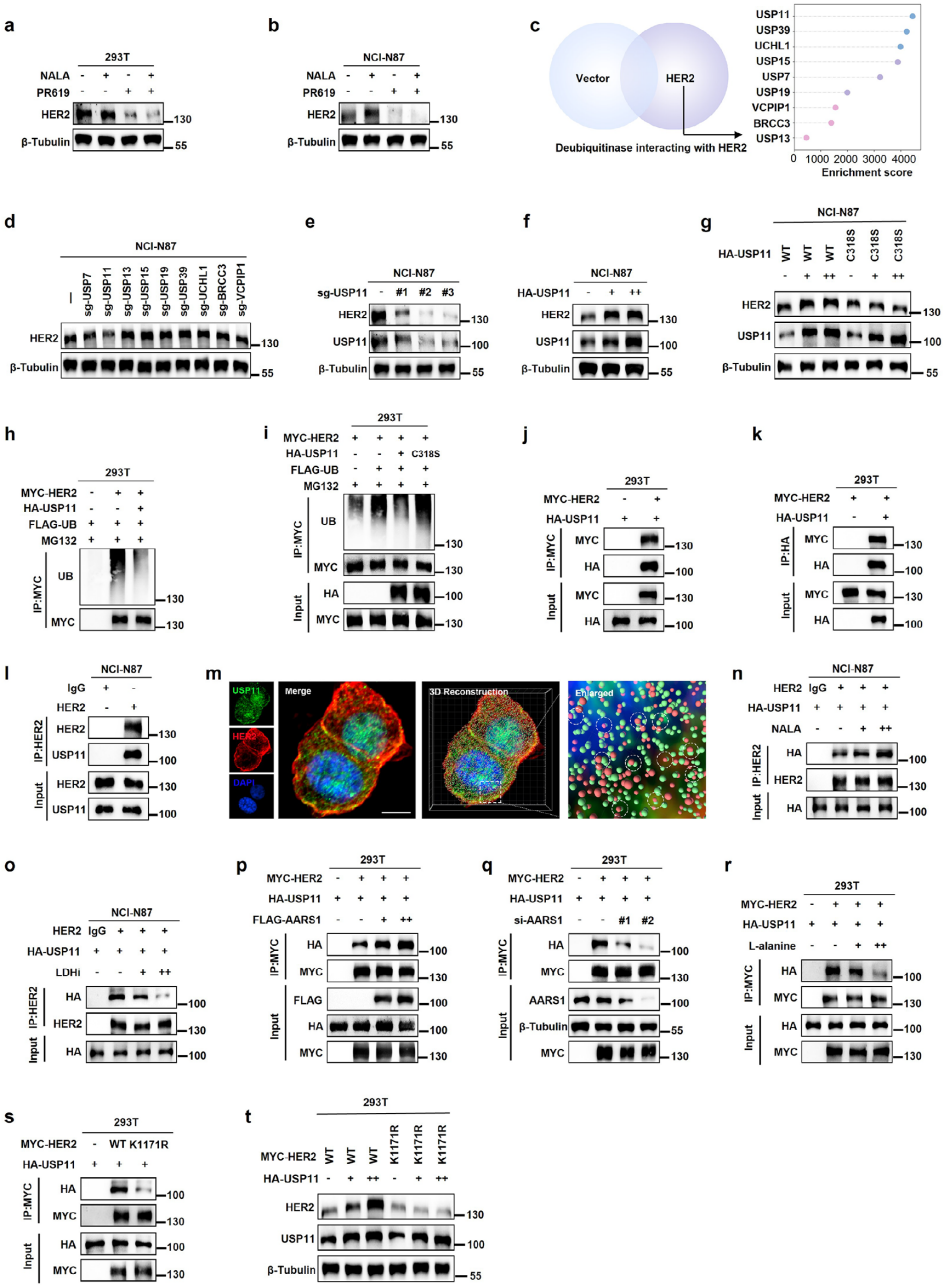
K1171 lactylation of HER2 promotes its interaction with USP11. (a,b) HER2 protein levels in 293T and NCI‐N87 cells treated with NALA and/or PR619 (10 µm). (c) Deubiquitinases (DUBs) interacting with HER2 identified by LC‐MS/MS analysis and ranked by enrichment score. (d) Knockout of the nine candidate DUBs to identify the key regulator of HER2 protein stability. (e) Western blot analysis of HER2 protein levels in NCI‐N87 cells following USP11 knockout. (f) Western blot analysis of HER2 protein levels in NCI‐N87 cells following USP11 overexpression. (g) HER2 protein levels following overexpression of USP11 WT and its inactive mutant. (h) Ubiquitination levels of HER2 in 293T cells following USP11 overexpression. (i) Ubiquitination levels of HER2 following overexpression of USP11 WT and its inactive mutant. (j,k) Exogenous Co‐IP assays confirming the interaction between HER2 and USP11 in 293T cells. (l) Endogenous Co‐IP assay confirming the interaction between HER2 and USP11 in NCI‐N87 cells. (m) IF staining showing the co‐localization of HER2 and USP11 in NCI‐N87 cells (scale bars, 5 µm). (n,o) Co‐IP analysis of the interaction between MYC‐HER2 and HA‐USP11 following NALA (n) and LDHi (o) treatment. (p–r) Co‐IP analysis of the interaction between HER2 and USP11 binding following AARS1 overexpression (p), AARS1 knockdown (q), and L‐alanine treatment (r). (s) Co‐IP analysis of USP11 binding to HER2 WT and the K1171R mutant. (t) Protein levels of HER2 WT and HER2 K1171R mutant following USP11 overexpression.

Co‐IP assays confirmed the interaction between HER2 and USP11 (Figure 3j–l). Meanwhile, IF staining revealed clear colocalization of the two proteins (Figure 3m). NALA treatment enhanced the binding between HER2 and USP11 in a dose‐dependent manner, while LDHi treatment weakened this interaction (Figure 3n,o). Similarly, AARS1 overexpression enhanced the binding between HER2 and USP11, while AARS1 knockdown diminished this interaction (Figure 3p,q). L‐alanine treatment likewise reduced the interaction between HER2 and USP11 (Figure 3r). Previous results demonstrated that the K1171R mutation significantly decreased HER2 lactylation. Here, we found that the HER2 K1171R mutant showed significantly reduced binding to USP11 compared with HER2 WT (Figure 3s). Notably, USP11 overexpression increased the protein levels of HER2 WT in a dose‐dependent manner, but had no effect on the HER2 K1171R mutant (Figure 3t).

### 2.4 AARS1‐mediated HER2 Lactylation Promotes Its Interaction With EGFR

NALA treatment significantly enhanced the binding between HER2 and EGFR at both endogenous and exogenous levels, whereas LDHi treatment attenuated this interaction (Figure 4a–d). Similarly, L‐alanine treatment significantly reduced the HER2‐EGFR interaction (Figure 4e). In vitro pull‐down assays using purified recombinant HER2 and EGFR proteins further confirmed that AARS1‐mediated lactylation of HER2 promotes the binding between the two proteins (Figure 4f). Consistently, IF staining demonstrated that NALA treatment upregulated HER2 expression and enhanced its colocalization with EGFR, whereas LDHi treatment downregulated HER2 expression and impaired this interaction (Figure 4g). Notably, NALA treatment failed to enhance the binding between the HER2 K1171R mutant and EGFR, underscoring the essential role of HER2 lactylation in HER2‐EGFR binding (Figure 4h). AARS1 overexpression significantly strengthened the binding between HER2 WT and EGFR, but had no effect on the HER2 K1171R mutant (Figure 4i). IF staining further confirmed that NALA treatment markedly increased lactylation at the K1171 site of HER2 and enhanced its binding to EGFR, whereas LDHi treatment significantly reduced HER2 K1171 lactylation and impaired HER2‐EGFR binding (Figure 4j).

**FIGURE 4:**
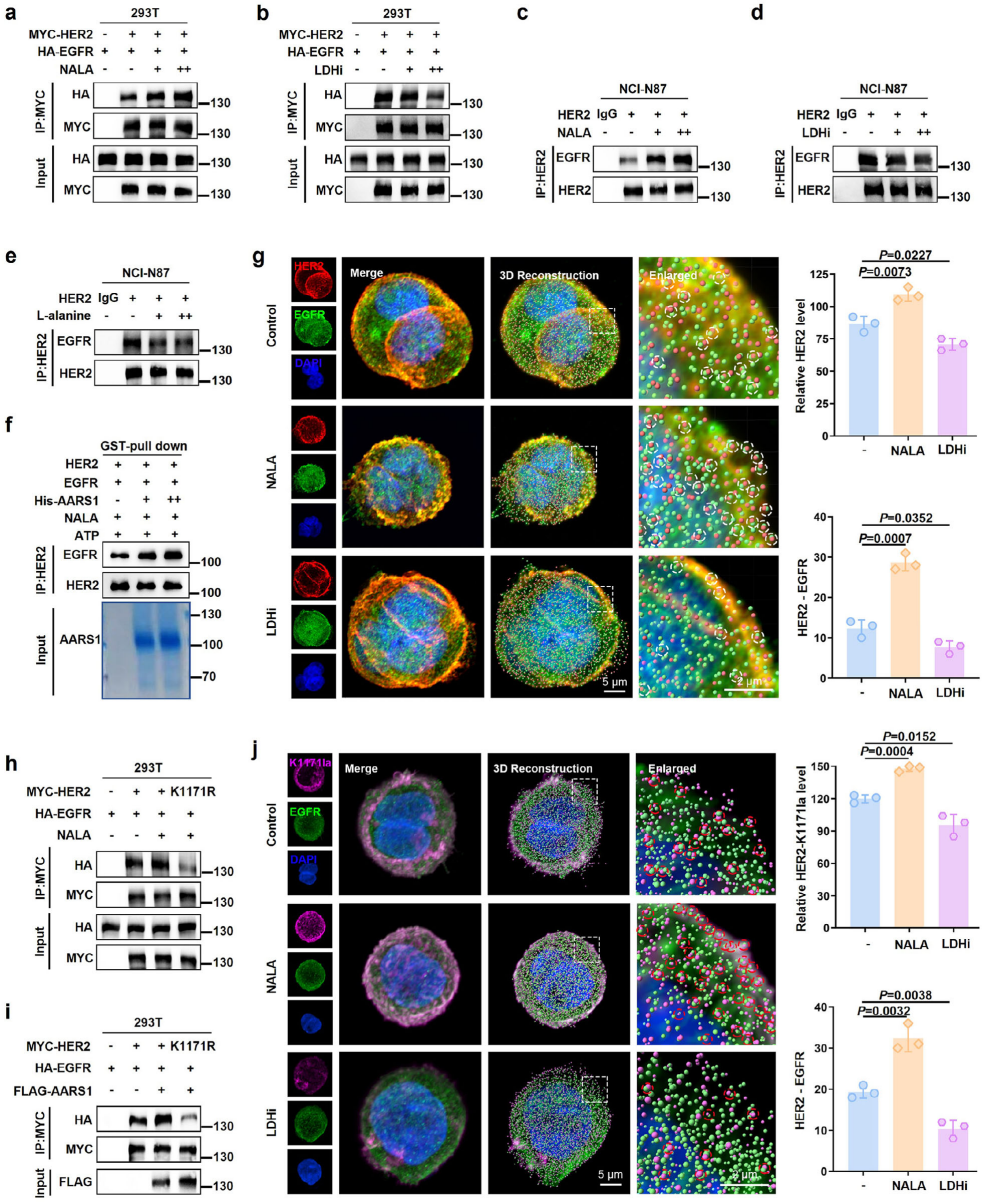
AARS1‐mediated HER2 lactylation promotes its interaction with EGFR. (a,b) The interaction between exogenously expressed HER2 and EGFR in 293T cells treated with NALA (a) and LDHi (b). (c–e) Co‐IP analysis of endogenous HER2‐EGFR binding in NCI‐N87 cells following NALA (c), LDHi (d), and L‐alanine treatment (e). (f) In vitro GST pull‐down assay showing the effect of AARS1‐induced HER2 lactylation on HER2‐EGFR binding. Coomassie blue staining of purified AARS1 used in the pull‐down assay. (g) Representative IF images and quantitative analysis of HER2 and EGFR in NCI‐N87 cells (*n* = 3) following NALA and LDHi treatment (scale bars, 5 µm). (h) Co‐IP analysis of EGFR binding to HER2 WT and HER2 K1171 mutant following NALA treatment. (i) Co‐IP analysis of EGFR binding to HER2 WT and HER2 K1171 mutant following AARS1 overexpression. (j) Representative IF images and quantitative analysis of HER2 K1171 lactylation and EGFR in NCI‐N87 cells (*n* = 3) following NALA and LDHi treatment (scale bars, 5 µm). *p*‐values were determined by Student's *t*‐test; ns, not significant. Data are presented as mean ± SD.

Given the structural and functional similarities among HER family members, we next examined whether AARS1‐mediated lactylation also regulates EGFR and HER3. Notably, EGFR was indeed lactylated, but its lactylation level was independent of AARS1 regulation (Extended Data Figure S3a,b). In contrast, no lactylation signal was detected on HER3 under the same experimental conditions (Extended Data Figure S3c).

### 2.5 Lactate Suppression Improves Trastuzumab Efficacy in Gastric Cancer

To evaluate the role of lactate in trastuzumab treatment for GC, we examined the effects of lactate suppression in vivo using multiple mouse models. According to the experimental design, mice were treated with trastuzumab, stiripentol (LDHi), or their combination. First, we established a HER2‐positive GC xenograft model using luciferase‐labeled NCI‐N87 cells (Figure 5a). Tumor bioluminescence was monitored weekly using the NightOWL II in vivo imaging system (Figure 5b). The combination therapy group exhibited significantly suppressed tumor growth, accompanied by a pronounced reduction in bioluminescence intensity compared with the monotherapy groups (Figure 5c,d). Flow cytometric analysis of dissected tumor tissues revealed that combination treatment with stiripentol and trastuzumab markedly reduced HER2 expression in vivo (Figure 5e,f). EdU incorporation assays indicated that the combination treatment significantly inhibited tumor cell proliferation (Figure 5g,h). IHC staining of tumor tissues further demonstrated a significant decrease in HER2 K1171 lactylation following combination therapy (Figure 5i,j). Notably, stiripentol exhibited no obvious systemic toxicity in mice, as evidenced by unchanged body weight and normal renal/hepatic function (Extended Data Figure S4a–e).

**FIGURE 5:**
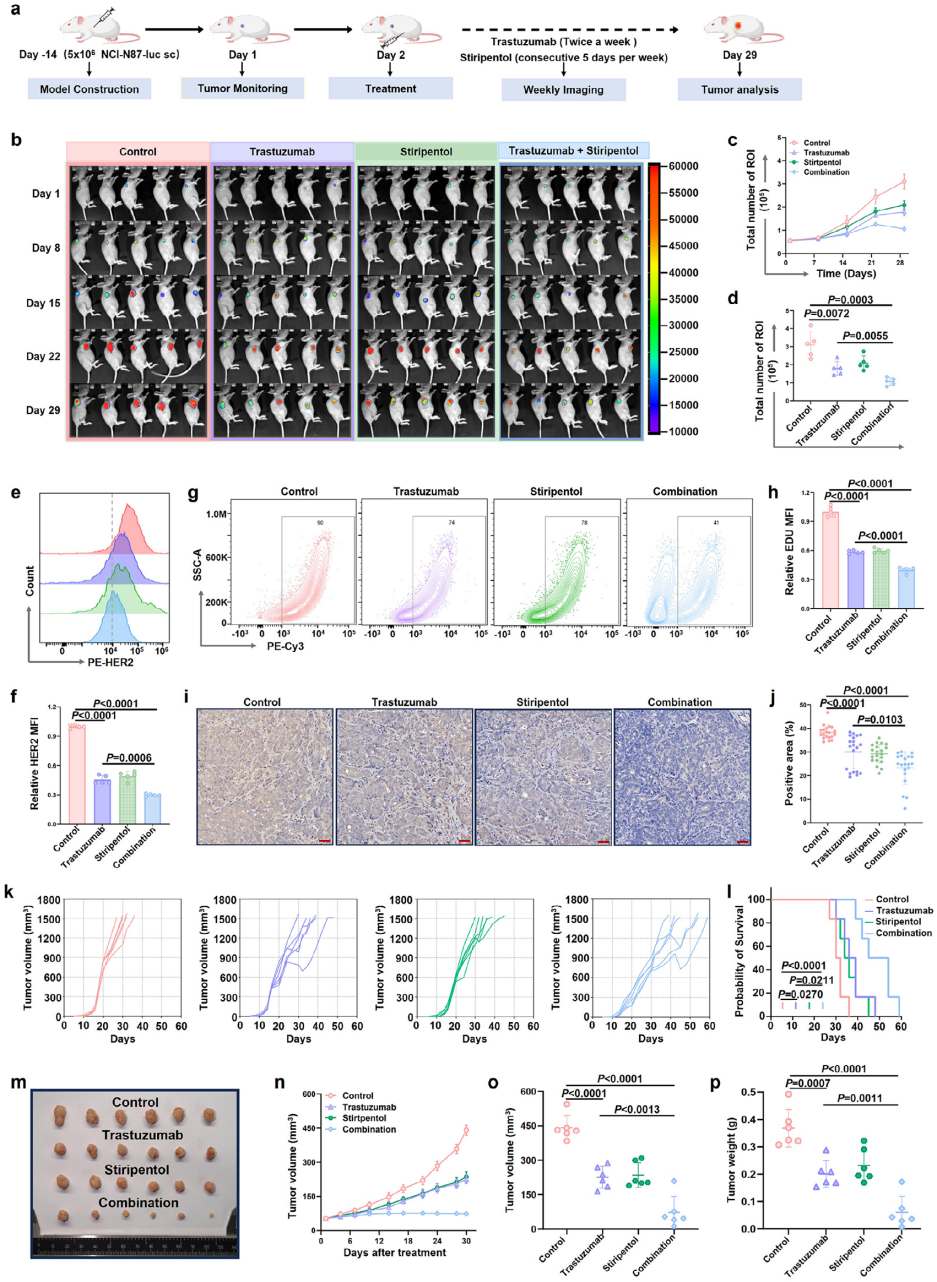
Lactate suppression improves trastuzumab efficacy in gastric cancer. (a) Schematic of luciferase‐labeled NCI‐N87 xenograft mouse model establishment. (b) Representative in vivo bioluminescence images of luciferase‐labeled gastric cancer xenografts (*n* = 5 per group). All images were acquired under identical exposure parameters and displayed on the same color scale (10,000–60,000). (c) Quantification of tumor burden by region of interest (ROI) analysis over the observation period. (d) Bioluminescence intensity of individual tumors at the end of treatment (*n* = 5). (e,f) Flow cytometric analysis and quantification of HER2 expression in mouse tumor tissues (*n* = 5). (g,h) Flow cytometric analysis and quantification of EdU incorporation in mouse tumor tissues (*n* = 5). (i,j) Representative immunohistochemical (IHC) staining images (scale bars, 50 µm) and quantitative analysis of HER2‐K1171la in mouse tumor tissues (*n* = 5, four random fields per tissue section were analyzed). (k,l) Tumor growth curves and Kaplan‐Meier survival analysis of all treatment groups in the NCI‐N87 gastric cancer xenograft model (*n* = 6; log‐rank test). (m) Representative images of dissected tumors from the HER2‐overexpressing AGS xenograft model. (n) Tumor volume monitoring in different treatment groups. (o) Volume measurement of dissected tumor (*n* = 6). (p) Weight measurement of dissected tumor (*n* = 6). *p*‐values were determined by two‐way ANOVA (c and n), mixed‐effects models (k), Student's *t*‐test (d, f, h, o, and p), or Mann‐Whitney *U* test (j); ns, not significant. Data are presented as mean ± SD.

To investigate the impact of lactate inhibition on overall survival, we established a separate HER2‐positive GC xenograft model using NCI‐N87 cells. A tumor volume of ≥ 1500 mm^3^ was defined as the survival endpoint for mice (Extended Data Figure S4f). Mice treated with the combination of trastuzumab and stiripentol exhibited significantly suppressed tumor growth and prolonged overall survival (Figure 5k,l).

We then validated these findings in a HER2‐overexpressing AGS xenograft model (Extended Data Figure S4g). Consistent with the NCI‐N87 model, combination therapy with stiripentol and trastuzumab significantly inhibited tumor growth, with smaller tumor volumes and lower tumor weights (Figure 5m–p). Flow cytometric analysis confirmed that the combination therapy markedly downregulated HER2 protein expression and suppressed tumor cell proliferation in vivo (Extended Data Figure S4h–k). Moreover, IHC staining demonstrated a significant decrease in HER2 K1171 lactylation in tumor tissues following combination therapy (Extended Data Figure S4l,m).

### 2.6 Inhibition of HER2 Lactylation Overcomes Trastuzumab Resistance in Gastric Cancer

By competing with lactate for AARS1 binding, L‐alanine potently antagonized AARS1‐mediated HER2 lactylation. We first established a HER2‐positive GC xenograft model using NCI‐N87 cells and treated the mice with trastuzumab, L‐alanine, or their combination (Extended Data Figure S5a). Tumors in the combination therapy group exhibited significantly slower growth, with correspondingly smaller tumor volumes and reduced tumor weights (Figure 6a–d). Flow cytometric analysis revealed marked downregulation of HER2 expression in tumor tissues and significant suppression of tumor cell proliferation following combination treatment (Extended Data Figure S5b–e). Additionally, IHC staining further confirmed a significant decrease in HER2 K1171 lactylation (Extended Data Figure S5f,g).

**FIGURE 6:**
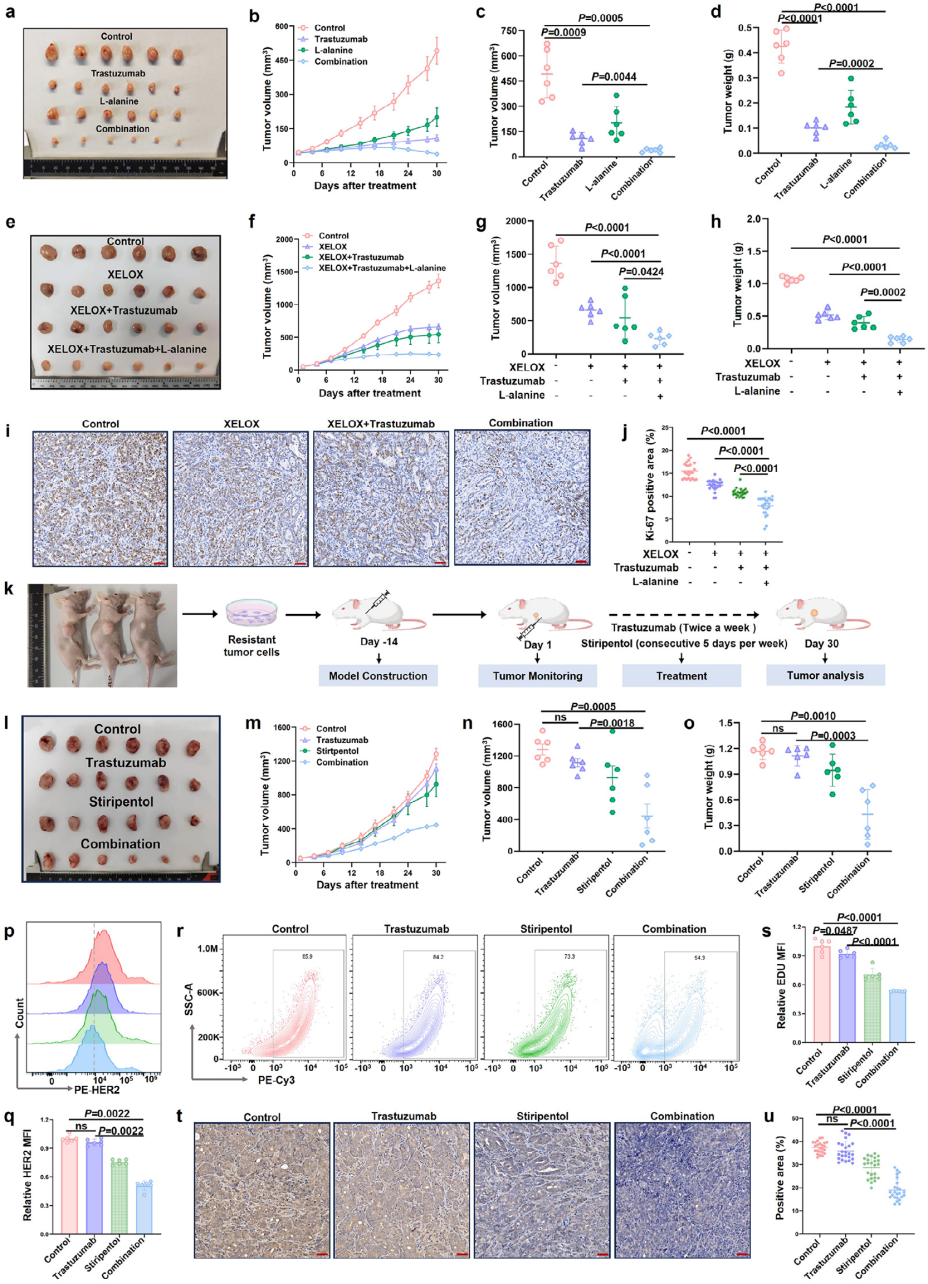
Inhibition of HER2 lactylation overcomes trastuzumab resistance in gastric cancer. (a) Representative images of dissected tumors from the NCI‐N87 xenograft model. (b) Tumor volume monitoring in different treatment groups. (c) End‐point volume of dissected tumors (*n* = 6). (d) End‐point weight of dissected tumors (*n* = 6). (e) Representative images of dissected tumors from the XELOX‐based combination therapy. (f) Tumor volume monitoring in different treatment groups. (g) End‐point volume of dissected tumors (*n* = 6). (h) End‐point weight of dissected tumors (*n* = 6). (i,j) Representative IHC staining images (scale bars, 50 µm) and quantification of Ki‐67 positive area in mouse tumor tissues (*n* = 6, four random fields per tissue section were analyzed). (k) Schematic illustration of the trastuzumab‐resistant gastric cancer mouse model establishment. (l) Representative images of dissected tumors from the resistant xenograft model. (m) Tumor volume curves in the trastuzumab‐resistant model. (n) End‐point volume in the resistant model (*n* = 6). (o) End‐point weight in the resistant model (*n* = 6). (p,q) Flow cytometric analysis of HER2 expression in tumors from the resistant model (*n* = 6). (r,s) Flow cytometric measurement of EdU incorporation in tumors from the resistant model (*n* = 6). (t,u) HER2‐K1171la IHC staining (scale bars, 50 µm) and quantification in the resistant model (*n* = 6, four random fields per tissue section were analyzed). XELOX, capecitabine plus oxaliplatin. *p*‐values were determined by two‐way ANOVA (b, f, and m), Student's *t*‐test (d, g, h, and n), Welch's‐*t* test (s) or Mann‐Whitney *U* test (j, o, q, and u); ns, not significant. Data are presented as mean ± SD.

To strengthen the clinical relevance of these findings, we established an additional HER2‐positive GC xenograft model and treated the mice with XELOX (capecitabine plus oxaliplatin) alone, trastuzumab plus XELOX, or the triple combination of trastuzumab, XELOX, and L‐alanine. Tumors in the triple‐combination group showed significantly slower growth, accompanied by smaller tumor volumes and reduced tumor weights (Figure 6e–h). Notably, the addition of L‐alanine did not cause any adverse effects on body weight and renal/hepatic function (Extended Data Figure S5h–l). Furthermore, IHC staining confirmed significant suppression of tumor cell proliferation following combination treatment (Figure 6i,j).

We isolated and digested tumor tissues from mice exhibiting poor response to trastuzumab, and used the resulting dissociated cells to establish a trastuzumab‐resistant GC xenograft model in nude mice (Figure 6k). The mice were randomly divided into four groups: control, trastuzumab, stiripentol, and a combination of stiripentol and trastuzumab. In the resistant model, trastuzumab monotherapy showed no significant inhibitory effect on tumor growth compared with the control group. In contrast, the combination of stiripentol and trastuzumab effectively restored sensitivity and suppressed tumor growth (Figure 6l,m). The combination therapy group exhibited significantly smaller tumor volumes and lower tumor weights compared with the other groups (Figure 6n,o). Flow cytometric analysis confirmed that stiripentol combined with trastuzumab downregulated HER2 expression and suppressed tumor cell proliferation in the resistant tumors (Figure 6p–s). Moreover, IHC staining revealed a marked reduction in HER2 K1171 lactylation following combination treatment (Figure 6t,u). Collectively, these findings demonstrate that the combination of stiripentol and trastuzumab effectively overcomes trastuzumab resistance in GC.

### 2.7 Targeting HER2 K1171 Lactylation With a Peptide Inhibitor Enhances Trastuzumab Sensitivity

Given that inhibition of AARS1 or LDH may affect multiple signaling pathways, a more specific strategy targeting HER2 K1171 lactylation is warranted. Based on the sequences flanking the HER2 K1171 site, we synthesized three short peptides fused with cell‐penetrating peptides (CPPs) (Figure 7a). Screening assays identified K1171‐peptide‐1# (termed K1171‐pe) as the most potent inhibitor of HER2 lactylation and protein expression (Figure 7b,c). A scrambled control peptide (termed K1171R‐pe), in which the K1171 residue was substituted with arginine (R), was also synthesized. Notably, only K1171‐pe, but not K1171R‐pe, significantly reduced HER2 lactylation levels (Figure 7d). Consistently, K1171‐pe markedly decreased HER2 protein expression, whereas K1171R‐pe had no effect (Figure 7e). Meanwhile, K1171‐pe treatment significantly inhibited the binding between endogenous HER2 and EGFR, whereas K1171R‐pe exerted no effect on HER2‐EGFR binding (Figure 7f). To further validate the target specificity of K1171‐pe, we examined its effect on intracellular lactate levels and global protein lactylation. Notably, cellular lactate concentrations remained unchanged following peptide treatment, suggesting that K1171‐pe does not broadly affect lactate production or transport (Extended Data Figure S6a). Furthermore, with the exception of HER2 protein, no significant alterations were detected in global lysine lactylation levels (Extended Data Figure S6b).

**FIGURE 7:**
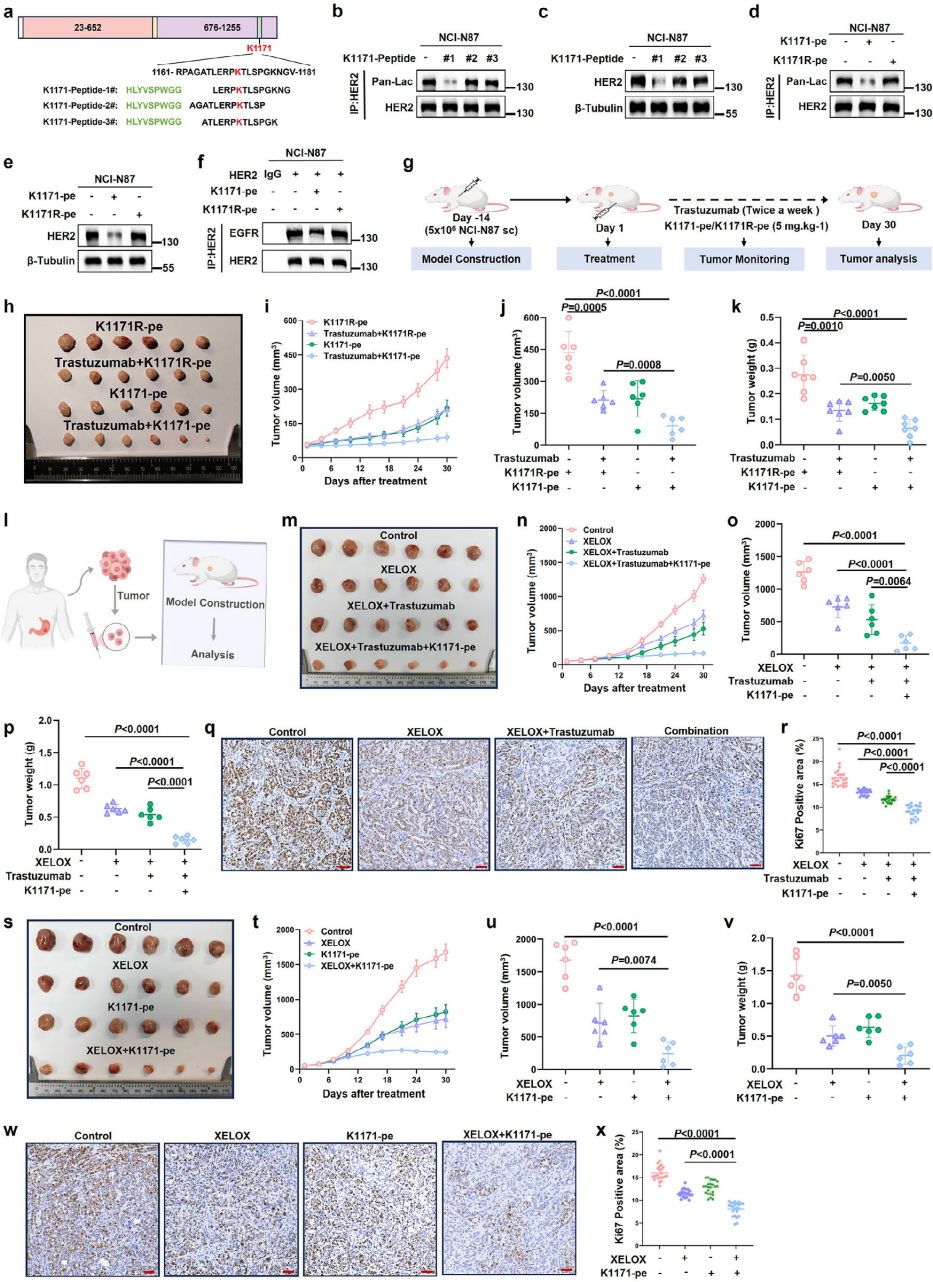
Targeting HER2 K1171 lactylation with a peptide inhibitor enhances trastuzumab sensitivity. (a) Schematic illustration of the designed peptide. Green represents the cell‐penetrating peptide (CPP), and red indicates the site for HER2 lactylation. (b,c) Western blot analysis of HER2 lactylation and protein expression in NCI‐N87 cells treated with the peptides. (d,e) Western blot analysis of HER2 lactylation and protein expression in NCI‐N87 cells treated with the peptide‐1# (K1171‐pe). (f) Co‐IP analysis of the binding between endogenous HER2 and EGFR in NCI‐N87 cells treated with K1171‐pe and control peptide (K1171R‐pe). (g) Experimental schematic of the NCI‐N87 xenograft mouse model. (h) Representative images of dissected tumors from the NCI‐N87 xenograft model. (i) Tumor volume monitoring in different treatment groups. (j) Endpoint volume of dissected tumors (*n* = 6). (k) Endpoint weight of dissected tumors (*n* = 6). (l) Experimental schematic of the patient‐derived xenograft (PDX) model. (m) Representative images of dissected tumors from the PDX model. (n) Tumor growth curves in the PDX model. (o) End‐point volume in the PDX model (*n* = 6). (p) End‐point weight in the PDX model (*n* = 6). (q,r) Representative IHC staining images (scale bars, 50 µm) and quantification of Ki‐67 positive area in mouse tumor tissues (*n* = 6, four random fields per tissue section were analyzed). (s) Representative images of dissected tumors from the HER2‐low expression model. (t) Tumor growth curves in the HER2‐low expression model. (u) End‐point tumor volume in the HER2‐low expression model (*n* = 6). (v) End‐point tumor weight in the HER2‐low expression model (*n* = 6). (w,x) Representative IHC staining images (scale bars, 50 µm) and quantification of Ki‐67 positive area in mouse tumor tissues (*n* = 6, four random fields per tissue section were analyzed). *p*‐values were determined by two‐way ANOVA (I, n, and t), Student's *t*‐test (j, k, o, p, u, and v), or Mann‐Whitney *U* test (h, r, and x); ns, not significant. Data are presented as mean ± SD.

To determine whether K1171‐pe enhances the therapeutic efficacy of trastuzumab in vivo, we established a HER2‐positive GC xenograft model using NCI‐N87 cells (Figure 7g). Notably, K1171‐pe, but not the control peptide K1171R‐pe, significantly enhanced the antitumor effect of trastuzumab (Figure 7h). Compared with other treatment groups, the combination of K1171‐pe and trastuzumab markedly suppressed tumor growth, reduced tumor volume, and decreased tumor weight (Figure 7i–k). IHC staining confirmed a significant reduction in HER2 K1171 lactylation following combination treatment (Extended Data Figure S6c,d). Furthermore, flow cytometric analysis of mouse tumor tissues confirmed that the combination therapy substantially reduced HER2 protein levels and suppressed tumor cell proliferation (Extended Data Figure S6e–g).

We further validated the effect of K1171‐pe on trastuzumab efficacy in GC using MFC cells with stable HER2 overexpression (Extended Data Figure S6h). Consistent with our previous findings, K1171‐pe significantly increased the sensitivity of GC cells to trastuzumab (Extended Data Figure S6i,j). Mice receiving the combination of K1171‐pe and trastuzumab exhibited significantly smaller tumor volumes and lower tumor weights compared with the other treatment groups (Extended Data Figure S6k,l).

To further validate the functional role of HER2 lactylation in vivo, we generated a patient‐derived xenograft (PDX) model from a HER2‐positive GC patient tumor (confirmed by HER2 IHC and H&E staining) (Figure 7l). Histological characterization of the PDX model and detailed patient information is provided in Table S4. Mice were randomly divided into four groups: control, XELOX alone, trastuzumab plus XELOX, and trastuzumab plus XELOX combined with K1171‐pe. Compared with XELOX alone or trastuzumab plus XELOX, the addition of K1171‐pe to trastuzumab plus XELOX exerted the most potent antitumor effect, resulting in significantly slower growth, smaller tumor volumes, and lower tumor weights compared with the other groups (Figure 7m–p). Importantly, including K1171‐pe in the combination regimen produced no detectable adverse effect on body weight (Extended Data Figure S6m). IHC staining further revealed significant downregulation of HER2 expression and suppressed tumor cell proliferation following the combination treatment (Figure 7q,r and Extended Data Figure S6n,o).

We next extended these findings to a HER2‐low expression context by establishing AGS cell‐derived xenograft models. Mice were treated with XELOX, K1171‐pe, or their combination. Compared with either monotherapy, the combination treatment resulted in significantly slower tumor growth, accompanied by smaller volumes and lower weights (Figure 7s–v). IHC staining confirmed significant suppression of tumor cell proliferation following combination treatment (Figure 7w,x). Notably, K1171‐pe showed no overt toxicity in mice, as assessed by body weight and renal/liver function (Extended Data Figure S6p–t).

Given the critical role of the tumor microenvironment (TME) in therapeutic responses, we also investigated whether HER2 lactylation exerts fundamental influences on microenvironmental cells. Intriguingly, K1171‐pe treatment reduced HER2 expression in lymphocytes. In contrast, the basal HER2 levels in fibroblasts and macrophages were extremely low, making precise quantification challenging (Extended Data Figure S7a,c,e). Functional analyses further demonstrated that K1171‐pe had no appreciable effect on lymphocyte effector function, fibroblast activation, or macrophage polarization (Extended Data Figure S7b,d,f). Taken together, these findings suggest that targeting HER2 K1171 lactylation may represent a promising strategy to enhance therapeutic efficacy in gastric cancer.

### 2.8 HER2 K1171 Lactylation is Increased in Trastuzumab‐Resistant Tumors and Associated With Poor Prognosis

To investigate the potential association between HER2 K1171 lactylation and trastuzumab resistance in GC, we performed IF staining on trastuzumab‐sensitive and trastuzumab‐resistant GC tissues using the site‐specific antibody HER2‐K1171la. The results revealed higher levels of HER2 K1171 lactylation in trastuzumab‐resistant tissues than in sensitive tissues (Figure 8a). Interestingly, protein expression of AARS1—the key “writer” responsible for HER2 lactylation—showed no significant difference between sensitive and resistant tumor tissues (Extended Data Figure S7g).

**FIGURE 8:**
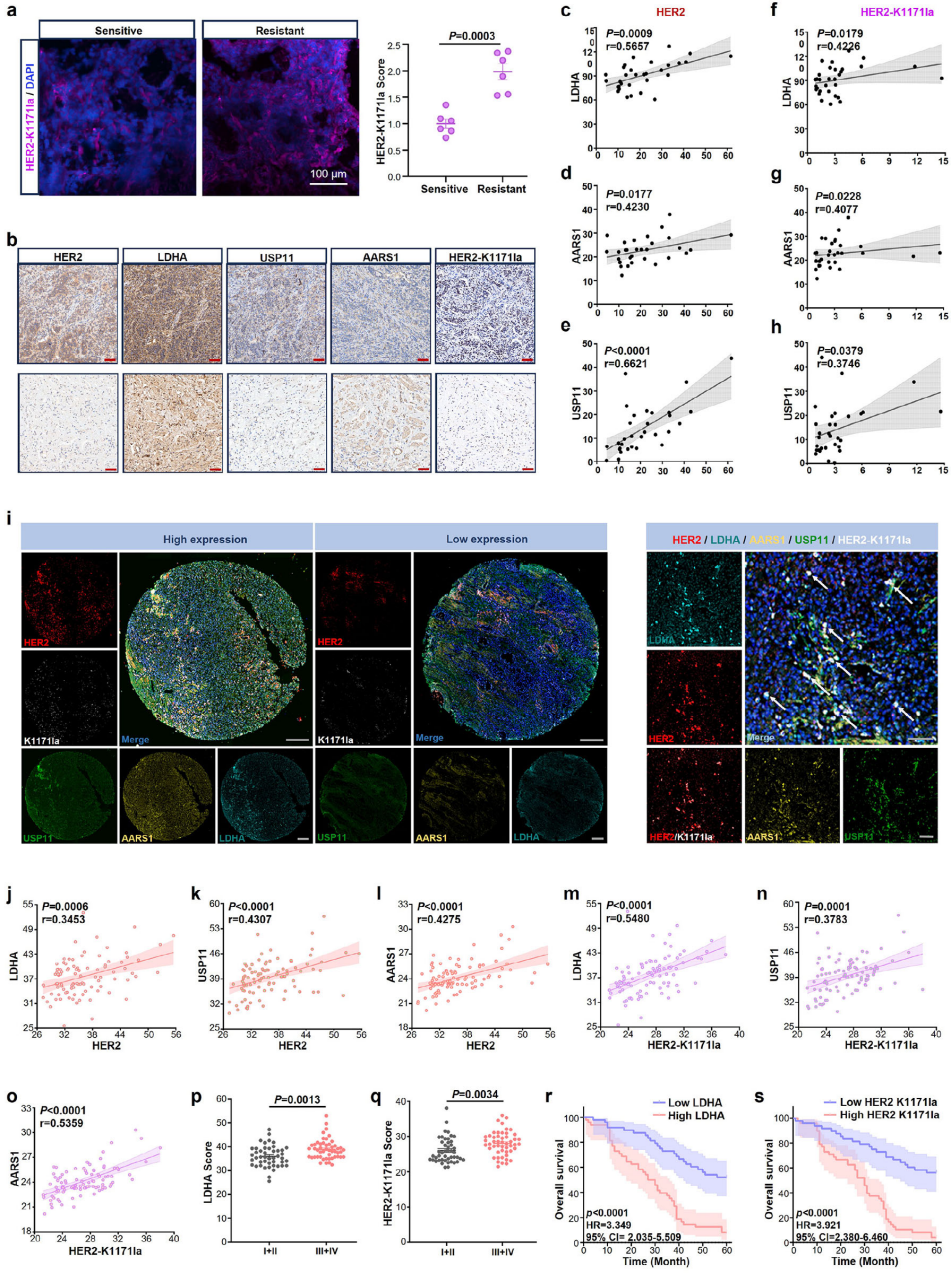
HER2 K1171 lactylation is increased in trastuzumab‐resistant tumors and associated with poor prognosis. (a) Representative IF images and quantitative analysis of HER2 K1171 lactylation in trastuzumab‐sensitive and trastuzumab‐resistant gastric cancer tissues (scale bars, 100 µm; *n* = 6). (b) Representative IHC staining images of human gastric cancer tissues (scale bars, 50 µm). (c–e) Correlation analyses of HER2 expression with LDHA expression (c), AARS1 expression (d), and USP11 expression (e) in gastric cancer tissues (*n* = 31; Spearman's rank correlation analysis). (f–h) Correlation analyses of HER2 lactylation with LDHA expression (f), AARS1 expression (g), and USP11 expression (h) in gastric cancer tissues (*n* = 31; Spearman's rank correlation analysis). (i) Multicolor IF staining of HER2, LDHA, AARS1, USP11, and HER2 lactylation in gastric cancer tissue microarrays (scale bars, 200 µm; 50 µm for magnified insets). (j–l) Correlation analyses of HER2 expression with LDHA expression (j), AARS1 expression (k), and USP11 expression (l) in the tissue microarray cohort (*n* = 96; Spearman's rank correlation analysis). (m–o) Correlation analyses of HER2 lactylation with LDHA expression (m), AARS1 expression (n), and USP11 expression (o) in the tissue microarray cohort (*n* = 96; Spearman's rank correlation analysis). (p) Quantification of LDHA expression in gastric cancer tissues across American Joint Committee on Cancer (AJCC) stages I‐IV (*n* = 96). (q) Quantification of HER2 lactylation across AJCC stages I‐IV (*n* = 96). (r,s) Kaplan‐Meier survival curves showing that high LDHA expression (r) and high HER2 K1171 lactylation (s) are associated with poor overall survival (*n* = 96, 48 patients per group; log‐rank test). High and low expression groups were defined based on the median cutoff values (LDHA score: 37.4612; HER2 K1171 lactylation: 26.9943). No cases were censored. *p*‐values were determined by Student's *t*‐test (a, d, f, and g) or Mann‐Whitney *U* test (p and q); ns, not significant. Data are presented as mean ± SD.

To evaluate the clinical relevance of HER2 expression and HER2 lactylation, we examined their correlations with LDHA expression, AARS1 expression, and USP11 expression. IHC staining was performed on 31 GC samples (Figure 8b). HER2 expression was positively correlated with LDHA expression, AARS1 expression, and USP11 expression (Figure 8c–e). Similarly, staining with the HER2‐K1171la antibody confirmed positive correlations of HER2 lactylation levels with both LDHA expression and AARS1 expression (Figure 8f–h). Notably, the positive correlation between HER2 K1171 lactylation and USP11 expression suggests that lactylated HER2 exhibits enhanced protein stability.

We further validated the clinical applicability of HER2 lactylation using GC tissue microarray combined with multiplex IF staining (Figure 8i). Consistently, our analyses showed positive correlations of LDHA expression, AARS1 expression, and USP11 expression with both HER2 lactylation and HER2 protein expression (Figure 8j–o). Notably, LDHA expression, HER2 lactylation, and AARS1 expression were significantly higher in advanced‐stage GC compared with early‐stage GC (Figure 8p,q and Extended Data Figure S7h). Furthermore, patients with high LDHA expression or high HER2 lactylation exhibited substantially poorer overall survival outcomes (Figure 8r,s).

## 3 Discussion

The Warburg effect, a well‐established metabolic hallmark of most cancers, is characterized by substantial lactate production [19, 20]. Beyond its conventional role as a metabolic byproduct, lactate is increasingly recognized as an important signaling molecule implicated in diverse pathophysiological processes [21, 22]. Lactylation, a recently identified post‐translational modification, has been shown to play a critical role in tumor progression and therapeutic responses [16, 23, 24]. Although limited studies have suggested that glucose metabolic reprogramming may contribute to trastuzumab resistance, the underlying mechanisms remain poorly understood [18, 25]. In the present study, we found that lactate levels were significantly elevated in trastuzumab‐resistant GC tumors, and that inhibition of HER2 lactylation markedly enhanced sensitivity to trastuzumab. These findings suggest a promising therapeutic strategy to improve the clinical efficacy of trastuzumab in GC.

In this study, we observed elevated lactate levels in trastuzumab‐resistant GC, and found that lactate significantly upregulated HER2 protein expression. It is well established that HER2 overexpression is associated with tumor resistance and poor prognosis [6, 26]. Liu et al. investigated the effects of HER2‐tyrosine kinase inhibitors (TKIs) on HER2‐positive GC cells, as well as their potential synergistic effects in combination with trastuzumab deruxtecan. Their findings demonstrated that irreversible HER2‐TKI treatment significantly reduced HER2 protein levels in GC cells. Mechanistically, irreversible HER2‐TKIs promoted HER2 internalization and degradation, thereby enhancing the therapeutic efficacy of trastuzumab deruxtecan [27]. In another study, the therapeutic potential of acetyltanshinone IIA was demonstrated in breast cancers resistant or non‐responsive to current HER2‐targeted therapies. Mechanistic analyses revealed that acetyltanshinone IIA promotes HER2 ubiquitination and subsequent proteasomal or lysosomal degradation, resulting in the downregulation of HER2 expression [7]. Taken together, these findings suggest that directly inducing HER2 degradation is an effective strategy to overcome trastuzumab resistance. Although HER2 protein stability is known to play a critical role in trastuzumab‐targeted therapy, the regulatory mechanisms underlying HER2 post‐translational modifications have not been fully elucidated.

Accumulating evidence has demonstrated the critical role of protein lactylation in driving resistance to cancer therapy [28, 29]. For example, NBS1 lactylation promotes homologous recombination‐mediated DNA repair and contributes to chemotherapy resistance [30]. In hepatocellular carcinoma, histone lactylation‐induced upregulation of MVP expression enhances immunotherapy resistance by inhibiting PD‐L1 degradation [31]. Elevated histone lactylation has also been detected in colorectal cancer (CRC) patients with bevacizumab resistance. In CRC cells, histone hyperlactylation induced by aerobic glycolysis promotes the transcription of RUBCNL, thereby enhancing autophagosome maturation and facilitating tumorigenesis and progression [32]. In this study, we identified AARS1‐mediated lactylation of HER2 at the K1171 site. This modification stabilized the HER2 protein by inhibiting USP11‐mediated proteasomal degradation. Notably, combining trastuzumab with either LDHi or L‐alanine—both inhibitors of HER2 lactylation—significantly improved therapeutic efficacy in HER2‐positive GC models. Furthermore, we designed and synthesized a peptide (K1171‐pe) that specifically inhibits HER2 K1171 lactylation. Treatment with K1171‐pe enhanced trastuzumab sensitivity in GC tumor models. Moreover, this peptide also exhibited significant combined antitumor effects in HER2‐low expression GC xenograft models. Taken together, our findings suggest that targeting HER2 lactylation represents a promising strategy to overcome trastuzumab resistance in HER2‐positive tumors.

We further observed that lactylation of HER2 at the K1171 site promoted its interaction with EGFR. Previous studies have established the critical role of HER2/EGFR heterodimerization in trastuzumab resistance [6, 8, 33]. Indeed, HER2 dimerization status has been proposed as a predictor of trastuzumab response, with higher HER2/EGFR heterodimer levels correlating with trastuzumab resistance and poor prognosis [34, 35]. HER2/EGFR heterodimerization was significantly enhanced in tumor models resistant to trastuzumab deruxtecan, in which these heterodimers impaired the efficiency of receptor clustering upon trastuzumab binding and attenuated HER2 endocytosis [12]. Notably, even in tumors with acquired trastuzumab resistance, HER2 and EGFR remain key oncogenic drivers in heterogeneous HER2‐positive breast cancer. Dual targeting of HER2 and EGFR can broadly inhibit HER2‐coordinated signaling and overcome trastuzumab resistance [36]. Consistent with these findings, our results demonstrate that reducing HER2 K1171 lactylation also significantly inhibits HER2‐EGFR binding.

In conclusion, our study reveals that lactate derived from the Warburg effect drives HER2 lactylation, a critical mediator contributing to trastuzumab resistance in GC. Mechanistically, HER2 lactylation stabilizes the HER2 protein by inhibiting USP11‐mediated proteasomal degradation, while concurrently enhancing the interaction between HER2 and EGFR. These findings identify a novel lactate‐mediated mechanism underlying trastuzumab resistance and provide important insights into the crosstalk between tumor metabolism and HER2‐targeted therapy resistance. Combining trastuzumab with clinically available LDHi agents—including stiripentol and L‐alanine—or with the K1171‐pe peptide potentiated therapeutic responses in HER2‐positive GC models. Notably, stiripentol—a clinically approved antiepileptic drug with validated LDH inhibitory activity—offers unique translational potential compared with other investigational LDH inhibitors lacking clinical validation, although its optimal dose and regimen for cancer therapy require further clinical investigation. Lactate‐targeting interventions may represent a promising strategy to overcome trastuzumab resistance and improve survival outcomes in GC patients. Furthermore, these findings also point to a potential therapeutic strategy for patients with HER2‐low expression.

Our study has some limitations. First, clinical GC specimens from patients with confirmed trastuzumab‐resistant are limited, and larger cohorts with longitudinal follow‐up are warranted for further validation. Second, although we demonstrated that HER2 lactylation promoted its interaction with EGFR, the precise molecular and structural basis underlying this modification require further investigation. Third, there is currently no effective amino acid substitution that mimics lysine lactylation. Hence, we cannot genetically generate HER2 K1171 lactylation‐mimetic mutant mice to study the roles of HER2 K1171 lactylation in tumor progression and therapeutic response in vivo. Finally, our in vivo experiments were conducted in immunodeficient mice—necessitated by the immune‐excluded phenotype of HER2‐positive GC tumors—which prevents direct assessment of HER2 lactylation effects on the tumor microenvironment.

## 4 Materials and Methods

### 4.1 Patient Samples

Tumor samples were obtained from patients diagnosed with HER2‐positive gastric cancer (GC) who underwent postoperative trastuzumab therapy at Tongji Hospital affiliated to Tongji University. HER2‐positivity was defined as immunohistochemistry (IHC) 3+ or IHC 2+ with HER2 gene amplification detected by fluorescence in situ hybridization (FISH) [2]. Trastuzumab resistance includes primary and acquired resistance: primary resistance was defined as progressive disease (PD) observed at the first imaging evaluation following first‐line trastuzumab therapy; acquired resistance was defined as initial remission or stabilization followed by PD during subsequent trastuzumab treatment [8]. This study was approved by the Medical Ethics Committee of Tongji Hospital affiliated to Tongji University (No. 2023‐004). In addition, GC tissue microarray for multiplex immunofluorescence (IF) staining analysis was obtained from Shanghai Outdo Biotech (No. YB M‐05‐01).

### 4.2 Untargeted Metabolomics Analysis

Untargeted liquid chromatography‐mass spectrometry (LC–MS/MS)‐based metabolomics was performed on gastric cancer tissue samples from trastuzumab‐sensitive and trastuzumab‐resistant patients, with 6 biological replicates per group (Thermo Fisher UltiMate 3000‐Exploris 480 Orbitrap). For metabolite extraction, frozen tumor tissues were mixed with 500 µL pre‐cooled methanol containing an internal standard and subjected to automated homogenization. The supernatant was collected through high‐speed centrifugation at 14,000 ×g for 20 min at 4°C, and then combined with methanol/chloroform for further extraction. After the second centrifugation, 200 µL of the supernatant was collected and lyophilized for subsequent LC‐MS/MS analysis. Quality control samples were prepared by mixing equal volumes from all experimental samples to monitor system stability throughout the analytical run. Chromatographic separation was performed on a Thermo Fisher UltiMate 3000 UHPLC system equipped with a Waters ACQUITY UPLC HSS T3 column (1.8 µm, 2.1 × 100 mm) maintained at 50°C. The mobile phase consisted of 0.1% formic acid in water and 0.1% formic acid in acetonitrile, delivered at a flow rate of 250 µL/min using a 15‐min gradient elution program. Mass spectrometry detection was conducted on a Thermo Fisher Exploris 480 Orbitrap mass spectrometer with a heated electrospray ionization source operating in both positive and negative ion modes. Full MS scan combined with data‐dependent acquisition MS/MS mode was applied, with mass range set at m/z 70–1050. Raw data were processed using Compound Discoverer software for peak alignment and metabolite annotation, followed by multivariate statistical analyses including principal component analysis, partial least squares discriminant analysis, and orthogonal partial least squares discriminant analysis to identify differentially expressed metabolites.

### 4.3 Measurement of Lactate

To measure lactate levels in tumor tissue, the tissue was homogenized with lysis buffer on ice. After centrifuging the samples at 12,000 g and 4°C for 10 min, the supernatants were collected. Then, lactate levels were measured using an L‐Lactate Assay Kit (Abcam ab65331).

### 4.4 Enzyme Linked Immunosorbent Assay (ELISA)

The concentration of HER2 was measured using a commercial ELISA kit (Elabscience E‐EL‐H6083) according to the product's instructions.

### 4.5 Quantitative Real‐Time PCR (qRT‐PCR)

Total RNA was extracted using the RNA Purification Kit and reverse transcribed into complementary DNA using a reverse transcription premix (#B0004D, EZBioscience). qRT‐PCR was performed on the Applied Biosystems 7300 Plus Sequence Detection System (Applied Biosystems). Expression levels of all target genes were normalized to the internal control. The qPCR primer sequences are listed in Table S5.

### 4.6 Western Blot and Co‐Immunoprecipitation (Co‐IP)

Cells were washed with phosphate‐buffered saline (PBS) and lysed in radioimmunoprecipitation assay (RIPA) buffer containing 1% protease inhibitor and 1% phosphatase inhibitor for 30 min at 4°C. After centrifugation at 12,000 rpm and 4°C for 10 min, the cell lysate supernatant was collected. Then, the supernatant was heated with 5× Native Gel Sample Loading Buffer (New Cell Molecular Biotech, WB3002) for 10 min at 100°C. The proteins were separated by SDS‐PAGE. For Co‐IP, cell lysate was incubated with various antibodies and protein A beads or anti‐FLAG/HA/MYC beads for 4–6 h at 4°C. Thereafter, the beads were washed three times with washing buffer and then subjected to Western blot analysis.

### 4.7 Flow Cytometric Analysis

The subcutaneous tumors were completely dissected and chopped into 1 mm^3^ pieces. Then, the tumor pieces were digested with collagenase and DNase for 30 min in a 37°C water bath. After filtering with a 70 µm cell strainer, single‐cell suspensions were collected. For cellular analysis, the cells were digested with trypsin, followed by resuspension in PBS containing 2% fetal bovine serum (FBS) for staining. Data were acquired on the CytoFLEX LX (Beckman Coulter) and analyzed with FlowJo software.

### 4.8 Immunofluorescence

Tissue samples from trastuzumab‐sensitive/resistant GC patients were fixed in 4% paraformaldehyde, and then dehydrated in 20% and 30% sucrose solutions. Tumor samples were then cut into 20 µm frozen sections using a cryostat. For immunofluorescence staining of human GC tissues, the sections were washed with PBS. For cellular immunofluorescence staining, the cells were washed with PBS, and fixed with 4% paraformaldehyde for 15 min at room temperature. Tissue sections/cells were permeabilized with Triton X‐100 (#P0096; Beyotime) in PBST (PBS with 0.1% Tween 20) for 15 min at room temperature. Sections/cells were blocked with blocking buffer for immunostaining (Beyotime, China) at room temperature for one hour, and then incubated with primary antibodies overnight at 4°C. Subsequently, sections/cells were incubated with secondary antibodies for one hour at room temperature. The fluorescence images were captured using a Nikon Ti2 confocal microscope (ECLIPSE Ti2, Nikon, Japan) and a Leica confocal microscope (Leica, Wetzlar, Germany). 3D reconstructions were completed with Imaris (Bitplane, version 9.1 Belfast, UK).

### 4.9 Mass Spectrometry Analysis

To identify HER2‐interacting proteins, NCI‐N87 cells were lysed using RIPA buffer supplemented with protease and phosphatase inhibitors. The cell lysates were incubated overnight at 4°C with anti‐HER2 antibody and isotype‐matched control IgG, respectively. Subsequently, the mixtures were incubated with Protein A/G agarose beads for 2 h at 4°C. The acquired MS/MS spectra were searched against the UniProt human proteome database. Relative protein abundance was calculated as the sum of all peptide intensities matched to each protein, and proteins were then ranked in descending order of their abundance values. For candidate protein screening, differences in protein abundance were first calculated between the HER2 IP group and the control IgG IP group. Proteins that were undetected in the control group but significantly enriched in the HER2 IP group were considered candidate interactors. From these candidates, proteins with reported or predicted functional roles in lysine lactylation were prioritized for further validation. Enrichment analysis was based on the functional annotation of the identified proteins. Stringent filtering criteria, including protein Q‐value < 0.01 and precursor Q‐value < 0.01, were applied during the database search stage to ensure high‐confidence protein identification.

Similarly, to identify HER2 lactylation sites, 293T cells stably expressing MYC‐tagged HER2 were treated with or without 50 mm sodium lactate (NALA) for 24 h. Cell lysates were prepared using RIPA buffer and purified using anti‐MYC agarose beads. The sediments were resolved on SDS‐PAGE and stained with Coomassie blue. The band corresponding to MYC‐HER2 was identified and sent for mass spectrometry analysis.

### 4.10 Peptide Synthesis

All synthetic peptides (Guoping Pharmaceutic Inc.) were used for both in vitro and in vivo experiments. The peptides were dissolved in PBS. For the in vitro experiments, a 10 mM stock solution of the peptides was prepared. For the in vivo experiments, K1171‐pe and K1171R‐pe were dissolved on ice for mouse injection.

### 4.11 Stable Cell Line Construction

To generate stable HER2‐overexpressing AGS/MFC cells, the HER2 lentivirus overexpression vector was transfected into 293T cells together with the packaging plasmids pMD2G and pSPAX2 at a ratio of 4:1:3. Supernatant containing the lentivirus was collected and used to infect gastric cancer cells. After 48 h of infection, the tumor cells were screened with puromycin (10 µg/mL).

### 4.12 Animal Tumor Model

All animal studies were performed in accordance with the ethical guidelines approved by the Department of Laboratory Animal Science of Tongji University (No. TJAB09624101). NCI‐N87 cells (5 × 10^6^), HER2‐overexpressing AGS cells (5 × 10^5^), and HER2‐overexpressing MFC cells (3 × 10^5^) were subcutaneously injected into the dorsal axillary region of 4–6‐week‐old female nude mice (JieSiJie Laboratory Animals).

For the patient‐derived xenograft (PDX) model, all experiments were strictly performed in accordance with ethical guidelines and approved by the Medical Ethics Committee of Tongji Hospital affiliated to Tongji University. Fresh tumor specimens were obtained from a patient with HER2‐positive GC who underwent surgical resection. Immediately after collection, the tumor tissues were preserved in cold physiological saline supplemented with 10% FBS and 1% penicillin‐streptomycin to maintain cell viability. Tumor samples were cut into 2–3 mm^3^ pieces and washed three times with cold physiological saline to remove residual blood and debris. The minced tumor fragments were then mixed with Matrigel at a 1:1 ratio and subcutaneously injected into 4–6‐week‐old female nude mice.

To better visualize tumor growth in mice, 5 × 10^6^ NCI‐N87 cells (volume: 0.1 mL) stably expressing luciferase were injected into the right side of nude mice (4–6‐week‐old). Two weeks later, in vivo bioluminescence was induced with D‐Luciferin substrate (Lablead, D12505). After anesthetizing mice with isoflurane, bioluminescence signals were detected using the IVIS Spectrum In Vivo Imaging System.

### 4.13 In Vivo Interventions

When the tumor volume reached 50 mm^3^, the mice were randomly divided into four groups (six mice per group): (1) control (100 µL PBS); (2) trastuzumab (20 mg/kg, intraperitoneal injection, twice a week) [37]; (3) stiripentol (150 mg/kg, intraperitoneal injection, consecutive 5 days per week) or L‐alanine (1.2% L‐alanine in water); (4) trastuzumab combined with stiripentol/L‐alanine.

For the animal experiments involving peptide treatment, mice were also randomly assigned to four groups: (1) K1171R‐pe (5 mg/kg, intraperitoneal injection, once a day); (2) K1171‐pe (5 mg/kg, intraperitoneal injection, once a day); (3) trastuzumab (20 mg/kg, intraperitoneal injection, twice a week) combined with K1171R‐pe (5 mg/kg, intraperitoneal injection, once a day); (4) trastuzumab (20 mg/kg, intraperitoneal injection, twice a week) combined with K1171‐pe (5 mg/kg, intraperitoneal injection, once a day).

Combination chemotherapy experiments were conducted in both HER2‐positive and HER2‐low expression GC models. In HER2‐positive xenografts, mice were randomly divided into four groups (*n* = 6 per group): (1) control (100 µL PBS); (2) XELOX alone (oxaliplatin: 10 mg/kg, administered intravenously on day 1; capecitabine: 359 mg/kg, once a day for 14 days) [38]; (3) XELOX plus trastuzumab (20 mg/kg, intraperitoneal injection, once a week); (4) XELOX plus trastuzumab combined with L‐alanine (1.2% L‐alanine in water, for NCI‐N87 models) or K1171‐pe (5 mg/kg, once a day, for PDX). In the HER2‐low expression GC model, mice were also randomized into four groups: (1) control (100 µL PBS); (2) XELOX; (3) K1171‐pe (5 mg/kg, intraperitoneal injection, once a day); (4) XELOX combined with K1171‐pe.

Tumor size was measured with a caliper, and tumor volume was calculated by the following formula: length×width^2^×0.523 [39]. According to the animal research protocol, mice were euthanized at the end of treatment or when the tumor volume reached 1,500 mm^3^. Tumor tissues were analyzed by IHC and flow cytometry.

### 4.14 Antibodies and Reagents

The following antibodies were from Proteintech: anti‐HER2 (18299‐1‐AP); anti‐β‐tubulin (10094‐1‐AP); anti‐6*His (66005‐1‐Ig); anti‐USP11 (10244‐1‐AP); anti‐AARS1 (17394‐1‐AP); anti‐EGFR (18986‐1‐AP). The anti‐LDHA (ET1608‐57) was from HUABIO. The following antibodies were from GNI GROUP LTD: anti‐HA (GNI#4510‐HA); anti‐Flag (GNI#4510‐FG); anti‐Myc (GNI#4510‐MC); anti‐HA‐HRP (GNI#4310‐HA); anti‐Flag‐HRP (GNI#4310‐FG); anti‐Myc‐HRP (GNI#4310‐MC). The anti‐pan‐Kla (PTM‐1401) was generated by PTM BIO. The anti‐Ub (3936) was from Cell Signaling Technology. The following antibody was from Abcam: anti‐HER2 (ab11710). The following antibody was from Biolegend: PE anti‐human CD340 (erbB2/HER‐2) Antibody (324406). The anti‐Myc‐Gel, anti‐HA‐Gel, and anti‐Flag‐Gel were purchased from GNI GROUP LTD. The EdU Imaging Kit was purchased from APExBIO (K1077). The anti‐HER2‐K1171la was generated by GL Biochem (Shanghai) Ltd. For reagents, MG132 (T2154), NH4Cl (12125‐02‐9), and PR619 (T1862) were purchased from Target Mol. Sodium L‐lactate (71718), L‐lactic acid (L1750), and sodium oxamate (O2751) were purchased from Sigma‐Aldrich. Trastuzumab and Stiripentol were purchased from MedChemExpress.

### 4.15 Quantification and Statistical Analysis

Statistical analysis was performed using GraphPad Prism 9.0 software, and data are presented as the mean ± SD. Normality was assessed using the Shapiro‐Wilk test. Pearson or Spearman correlation coefficients were used for correlation analyses, depending on whether the data were normally distributed. Survival curves were estimated using the Kaplan–Meier method, and groups were compared using the log‐rank test. For all experiments involving four groups, one‐way ANOVA was used for single‐time‐point comparisons, and two‐way ANOVA was used for experiments with a two‐factor design. Longitudinal tumor growth data were analyzed using two‐way repeated‐measures ANOVA or mixed‐effects models. Post hoc pairwise comparisons were performed only when the overall F‐test was significant, with Tukey's or Bonferroni correction applied for multiple comparisons. A *p*‐value < 0.05 was considered statistically significant.

## Author Contributions

Meng Qian, Tongguan Tian, and Zhenxiang Wang played major roles in designing and doing the experiments, analysing the results, and organising the figures. Zhiqian Hu provided human GC samples and demonstrated the clinical relevance. Huihui Sun, Ming Zong, Ying Chen, Jie Xiong, and Ye Chen helped to conduct the experiments. Meng Qian, Yanping Xu, and Shuchang Xu wrote the manuscript. Tianming Li, Yanping Xu, and Shuchang Xu conceived and designed the study. Shuchang Xu supervised the study. The order of co‐first authors was determined by the volume of work each contributed to the study.

## Conflicts of Interest

The authors declare no conflicts of interest.

## Supporting information




**Supporting File 1**: advs77955‐sup‐0001‐SuppMat.docx

**Supporting File 2**: advs77955‐sup‐0002‐Table1.xlsx

**Supporting File 3**: advs77955‐sup‐0003‐Table2.xlsx

**Supporting File 4**: advs77955‐sup‐0004‐Table3.xlsx

**Supporting File 5**: advs77955‐sup‐0005‐Table4.xlsx

**Supporting File 6**: advs77955‐sup‐0006‐Table5.xlsx

## Data Availability

All data underlying this study are provided in the article and its Supplementary Information. Additional data related to this study can be obtained from the corresponding author upon reasonable request.

## References

[1] F. Bray , M. Laversanne , and H. Sung , et al., “Global CANCER Statistics 2022: GLOBOCAN Estimates of Incidence and Mortality Worldwide for 36 Cancers in 185 Countries,” CA: A Cancer Journal for Clinicians 74 (2024): 229–263, 10.3322/caac.21834. 38572751

[2] J. A. Ajani , T. A. D'Amico , and D. J. Bentrem , “Gastric Cancer, Version 2.2025, NCCN Clinical Practice Guidelines In Oncology,” Journal of the National Comprehensive Cancer Network 23 (2025): 169–191, 10.6004/jnccn.2025.0022. 40341199

[3] L. Jones , D. Cunningham , and N. Starling , et al., “HER‐2 Directed Therapies Across Gastrointestinal Tract Cancers—A New Frontier,” Cancer Treatment Reviews 129 (2024): 102789, 10.1016/j.ctrv.2024.102789. 38959629

[4] T. Sheng , R. Sundar , and S. Srivastava , et al., “Spatial Profiling of Patient‐Matched HER2 Positive Gastric Cancer Reveals Resistance Mechanisms to Targeted Therapy,” Gut 75 (2025): 733–747, 10.1136/gutjnl-2024-334667. 41167802

[5] Y. Zhang , “The Root Cause of Drug Resistance in HER2‐Positive Breast Cancer and the Therapeutic Approaches to Overcoming the Resistance,” Pharmacology & Therapeutics 218 (2021): 107677, 10.1016/j.pharmthera.2020.107677. PMC7855784 32898548

[6] Z. Wang , Z. Li , J. Högström , et al., “Dual Membrane Receptor Degradation Via Folate Receptor Targeting Chimera,” Nature Communications 16 (2025): 8804, 10.1038/s41467-025-63882-5. PMC12491540 41038820

[7] B. Huang , W. K. Yip , N. Wei , and K. Q. Luo , “Acetyltanshinone IIA is More Potent Than Lapatinib in Inhibiting Cell Growth and Degrading HER2 Protein in Drug‐Resistant HER2‐Positive Breast Cancer Cells,” Cancer Letters 490 (2020): 1–11, 10.1016/j.canlet.2020.06.010. 32585412

[8] J. Yoon and D.‐Y. Oh , “HER2‐Targeted Therapies Beyond Breast Cancer — An Update,” Nature Reviews Clinical Oncology 21 (2024): 675–700, 10.1038/s41571-024-00924-9. 39039196

[9] N. E. Weisser , M. Sanches , E. Escobar‐Cabrera , et al., “An Anti‐HER2 Biparatopic Antibody That Induces Unique HER2 Clustering and Complement‐Dependent Cytotoxicity,” Nature Communications 14 (2023): 1394, 10.1038/s41467-023-37029-3. PMC10011572 36914633

[10] X. Liu , Y. Song , P. Cheng , B. Liang , and D. Xing , “Targeting HER2 in Solid Tumors: Unveiling the Structure and Novel Epitopes,” Cancer Treatment Reviews 130 (2024): 102826, 10.1016/j.ctrv.2024.102826. 39270365

[11] M. R. Strickland and S. J. Klempner , “Dual HER2 Inhibition: Is Two Better Than One?,” Med 5 (2024): 1191–1193, 10.1016/j.medj.2024.07.008. 39395400

[12] A. Gupta , F. Michelini , H. Shao , et al., “EGFR‐Directed Antibodies Promote HER2 ADC Internalization and Efficacy,” Cell Reports Medicine 5 (2024): 101792, 10.1016/j.xcrm.2024.101792. PMC11604483 39437778

[13] M. G. Vander Heiden , L. C. Cantley , and C. B. Thompson , “Understanding the Warburg Effect: The Metabolic Requirements of Cell Proliferation,” Science 324 (2009): 1029–1033, 10.1126/science.1160809. PMC2849637 19460998

[14] J. Dai , X. Lu , C. Zhang , et al., “NNMT Promotes Acquired EGFR‐TKI Resistance by Forming EGR1 and lactate‐Mediated Double Positive Feedback loops in Non‐Small Cell Lung Cancer,” Molecular Cancer 24 (2025): 79, 10.1186/s12943-025-02285-y. PMC11909984 40089784

[15] M. Jin , B. Huang , X. Yang , et al., “Lactylation of XLF Promotes Non‐Homologous End‐Joining Repair and Chemoresistance in Cancer,” Molecular Cell 85 (2025): 2654–2672.e7, 10.1016/j.molcel.2025.06.019. 40680721

[16] J. Chen , Z. Huang , Y. Chen , et al., “Lactate and Lactylation in Cancer,” Signal Transduction and Targeted Therapy 10 (2025): 38, 10.1038/s41392-024-02082-x. PMC11814237 39934144

[17] X. Ji and L. Xia , “Lactate and Lactylation in Cancer: Drivers of Immune Suppression and Microenvironmental Reprogramming,” Experimental Hematology & Oncology 14 (2025): 128, 10.1186/s40164-025-00719-3. PMC12570437 41152975

[18] J. Wang , Q. Huang , X. Hu , et al., “Disrupting Circadian Rhythm via the PER1–HK2 Axis Reverses Trastuzumab Resistance in Gastric Cancer,” Cancer Research 82 (2022): 1503–1517, 10.1158/0008-5472.Can-21-1820. PMC9662874 35255118

[19] M. V. Liberti and J. W. Locasale , “The Warburg Effect: How Does it Benefit Cancer Cells?,” Trends in Biochemical Sciences 41 (2016): 211–218, 10.1016/j.tibs.2015.12.001. PMC4783224 26778478

[20] L. Zhao , H. Cui , Y. Li , Y. Ye , and Z. Shen , “Metabolite to Modifier: Lactate and Lactylation in the Evolution of Tumors,” MedComm 6 (2020): 70413, 10.1002/mco2.70413. PMC12497687 41059490

[21] M. Ajam‐Hosseini , R. Heydari , M. Rasouli , et al., “Lactic Acid in Macrophage Polarization: A Factor in Carcinogenesis and a Promising Target for Cancer Therapy,” Biochemical Pharmacology 222 (2024): 116098, 10.1016/j.bcp.2024.116098. 38431231

[22] C. Hayes , C. L. Donohoe , M. Davern , and N. E. Donlon , “The Oncogenic and Clinical Implications of Lactate Induced Immunosuppression in the Tumour Microenvironment,” Cancer Letters 500 (2021): 75–86, 10.1016/j.canlet.2020.12.021. 33347908

[23] D. Zhang , Z. Tang , H. Huang , et al., “Metabolic Regulation of Gene Expression by Histone Lactylation,” Nature 574 (2019): 575–580, 10.1038/s41586-019-1678-1. PMC6818755 31645732

[24] Y. Sun , H. Wang , Z. Cui , et al., “Lactylation in Cancer Progression and Drug Resistance,” Drug Resistance Updates 81 (2025): 101248, 10.1016/j.drup.2025.101248. 40287994

[25] J. Liu , C. Pan , L. Guo , et al., “A New Mechanism of Trastuzumab Resistance in Gastric Cancer: MACC1 Promotes the Warburg Effect via Activation of the PI3K/AKT Signaling Pathway,” Journal of Hematology & Oncology 9 (2016): 76, 10.1186/s13045-016-0302-1. PMC5007850 27581375

[26] A. G. Waks , O. Martínez‐Sáez , P. Tarantino , et al., “Dual HER2 Inhibition: Mechanisms of Synergy, Patient Selection, and Resistance,” Nature Reviews Clinical Oncology 21 (2024): 818–832, 10.1038/s41571-024-00939-2. 39271787

[27] J. Liu , T. Zhu , R. Zhao , W. Ren , F. Zhao , and J. Liu , “Elucidating Molecular Mechanisms and Therapeutic Synergy: Irreversible HER2‐TKI plus T‐Dxd for Enhanced Anti‐HER2 Treatment of Gastric Cancer,” Gastric Cancer 27 (2024): 495–505, 10.1007/s10120-024-01478-6. PMC11016512 38386239

[28] Z. R. Xue , Y. Y. Xin , and W. L. Jin , “Exploiting Metabolic Vulnerabilities in Cancer: From Mechanisms to Therapeutic Opportunities,” Cancer Letters 634 (2025): 218067, 10.1016/j.canlet.2025.218067. 41016676

[29] X. Hou , Z. Hong , H. Zeng , et al., “Lactylation in Cancer Biology: Unlocking New Avenues for Research and Therapy,” Cancer Communications 45 (2025): 1367–1406, 10.1002/cac2.70054. PMC12629867 40831053

[30] H. Chen , Y. Li , H. Li , et al., “NBS1 Lactylation is Required for Efficient DNA Repair and Chemotherapy Resistance,” Nature 631 (2024): 663–669, 10.1038/s41586-024-07620-9. PMC11254748 38961290

[31] S. Liu , Y. Pan , W. Liu , et al., “Lactylation‐Driven MVP Upregulation Boosts Immunotherapy Resistance by Inhibiting PD‐L1 Degradation in Hepatocellular Carcinoma,” Journal for ImmunoTherapy of Cancer 13 (2025): 012230, 10.1136/jitc-2025-012230. PMC12458742 40983342

[32] W. Li , C. Zhou , L. Yu , et al., “Tumor‐Derived Lactate Promotes Resistance to Bevacizumab Treatment by Facilitating Autophagy Enhancer Protein RUBCNL Expression Through Histone H3 Lysine 18 Lactylation (H3K18la) in Colorectal Cancer,” Autophagy 20 (2024): 114–130, 10.1080/15548627.2023.2249762. PMC10761097 37615625

[33] W. Gao , C. Zhang , X. Yang , et al., “Synergistic SuFEx‐Driven Covalent Cancer Therapy Using a HER2/EGFR Bivalent Affinity Protein Drug,” Angewandte Chemie International Edition 65 (2025): 17155, 10.1002/anie.202517155. 41178218

[34] R. Ghosh , A. Narasanna , S. E. Wang , et al., “Trastuzumab has Preferential Activity Against Breast Cancers Driven by HER2 Homodimers,” Cancer Research 71 (2011): 1871–1882, 10.1158/0008-5472.Can-10-1872. PMC3221734 21324925

[35] Y. Zhang , “Targeting Epidermal Growth Factor Receptor for Cancer Treatment: Abolishing Both Kinase‐Dependent and Kinase‐Independent Functions of the Receptor,” Pharmacological Reviews 75 (2023): 1218–1232, 10.1124/pharmrev.123.000906. PMC10595022 37339882

[36] L. Yang , A. Bhattacharya , D. Peterson , et al., “Targeted Dual Degradation of HER2 and EGFR Obliterates Oncogenic Signaling, Overcomes Therapy Resistance, and Inhibits Metastatic Lesions in HER2‐Positive Breast Cancer Models,” Drug Resistance Updates 74 (2024): 101078, 10.1016/j.drup.2024.101078. PMC11070302 38503142

[37] X. Hu , Z. Ma , B. Xu , et al., “Glutamine Metabolic Microenvironment Drives M2 Macrophage Polarization to Mediate Trastuzumab Resistance in HER2‐Positive Gastric Cancer,” Cancer Communications 43 (2023): 909–937, 10.1002/cac2.12459. PMC10397568 37434399

[38] S. Harada , M. Yanagisawa , S. Kaneko , et al., “Superior Antitumor Activity of Trastuzumab Combined With Capecitabine Plus Oxaliplatin in a Human Epidermal Growth Factor Receptor 2‐Positive Human Gastric Cancer Xenograft Model,” Molecular and Clinical Oncology 3 (2015): 987–994, 10.3892/mco.2015.609. PMC4534867 26623038

[39] T. Tian , X. Xie , W. Yi , et al., “FBXO38 mediates FGL1 Ubiquitination and Degradation to Enhance Cancer Immunity and Suppress Inflammation,” Cell Reports 42 (2023): 113362, 10.1016/j.celrep.2023.113362. 37938970

