# Supplementary material for: Lactylation‐Mediated HER2 Stabilization Promotes Trastuzumab Resistance in Gastric Cancer
Source: Adv Sci (Weinh). 2026 Oct 11:e77955. Online ahead of print. doi: 10.1002/advs.77955 (PMC13655267; doi:10.1002/advs.77955)
Supplement: Supplementary file 1 [file ADVS-9999-e77955-s004.docx]

**Figure. S1 Lactate promotes trastuzumab resistance and stabilizes HER2 expression in gastric cancer. a-b**, HER2 protein levels in 293T cells following treatment with NALA (25 or 50 mM) and lactate (10 or 20 mM). **c-d,** HER2 protein levels in 293T cells following treatment with sodium oxamate (10 or 20 mM) and stiripentol (100 or 200 μM). **e-p,** mRNA expression levels of HER2 in 293T (e-h), AGS (i-l), and NCI-N87 cells (m-p) following lactate supplementation or LDHi treatment. **q,** Flow cytometric analysis of HER2 expression in 293T cells treated with NALA or LDHi (n = 3). **r,** Representative immunofluorescence (IF) staining of pan-lactylation and HER2 in gastric cancer tissues (scale bars, 20 μm). **s,** HER2 protein levels in NCI-N87 cells treated with MG132 (10 μM) or NH4Cl (20 mM). *p*-values were determined by Student’s *t*-test; ns, not significant. Data are presented as mean ± SD.

**Figure. S2 AARS1-mediated HER2 K1171 lactylation enhances its protein stability. a,** Exogenous co-immunoprecipitation (Co-IP) assay confirming the interaction between HER2 and AARS1 in 293T cells. **b-d,** Spectra of HER2 lactylation sites at K747, K762, and K1171 identified by LC-MS/MS. **e,** Validation of the HER2 K1171 lactylation-specific antibody by Western blot. **f,** Dot blot assay confirming the specificity of the HER2 K1171 lactylation antibody. **g,** HER2 mRNA levels in NCI-N87 cells following AARS1 overexpression in NCI N87 cells (n = 3). **h,** HER2 mRNA levels in NCI-N87 cells following AARS1 knockdown (n = 3). **i,** HER2 protein levels in NCI-N87 cells with AARS1 overexpression and/or MG132 treatment. *p*-values were determined by Student’*s* *t*-test; ns, not significant. Data are presented as mean ± SD.

**Figure. S3 Lactylation of HER family members EGFR and HER3.** **a,** Co-IP analysis of EGFR lactylation in 293T cells following NALA and LDHi treatment. **b,** Co-IP analysis of EGFR lactylation in 293T cells following AARS1 overexpression. **c,** Co-IP analysis of HER3 lactylation in 293T cells following NALA and LDHi treatment.

**Figure. S4 Inhibition of HER2 lactylation sensitizes gastric cancer to trastuzumab. a,** Body weight monitoring in NCI-N87 xenograft model. **b-e,** Serum biochemical analyses of ALT (b), AST (c), BUN (d), and Cr (e) in mouse treated with stiripentol (n = 5). **f,** Schematic illustrating the establishment of NCI-N87 xenograft model for survival analysis. **g,** Schematic of the HER2-overexpressing AGS xenograft model. **h-i,** Flow cytometric analysis of HER2 expression in mouse tumor tissues (n = 6). **j-k,** Flow cytometric measurement of EdU incorporation in mouse tumor tissues (n = 6). **l-m,** Representative immunohistochemical (IHC) staining images (scale bars, 50 μm) and quantification of HER2-K1171la in mouse tumor tissues (n = 6, four random fields per tissue section were analyzed). ALT, alanine aminotransferase; AST, aspartate aminotransferase; BUN, blood urea nitrogen; Cr: creatinine. *p*-values were determined by Student’s *t*-test (b-e, I, and m) or Welch’s-*t* test (k); ns, not significant. Data are presented as mean ± SD.

**Figure. S5 Inhibition of HER2 lactylation overcomes trastuzumab resistance in gastric cancer. a,** Schematic illustration of the NCI-N87 xenograft model. **b-c,** Flow cytometric analysis of HER2 expression in mouse tumors (n = 6). **d-e,** Flow cytometric analysis of EdU incorporation in mouse tumors (n = 6). **f-g,** Representative IHC staining images (scale bars, 50 μm) and quantification of HER2-K1171la in mouse tumor tissues (n = 6, four random fields per tissue section were analyzed). **h,** Body weight monitoring in different treatment groups. **i-l,** Serum biochemical analyses of ALT (i), AST (j), BUN (k), and Cr (l) in mouse treated with K1171-pe (n = 6). ALT, alanine aminotransferase; AST, aspartate aminotransferase; BUN, blood urea nitrogen; Cr: creatinine. *p*-values were determined by Student’s *t*-test (c and i-l), Mann-Whitney *U* test (e), or Welch’s-*t* test (g); ns, not significant. Data are presented as mean ± SD.

**Figure. S6 Targeting HER2 K1171 lactylation with a peptide inhibitor enhances trastuzumab sensitivity. a,** Measurement of lactate levels in NCI-N87 cells (n = 3). **b,** Western blot analysis of global lysine lactylation (Pan-kla) in NCI-N87 cells. **c-d,** Representative IHC staining images (scale bars, 50 μm) and quantitative analysis of HER2-K1171la in the NCI-N87 model (n = 6, four random fields per tissue section were analyzed). **e-f,** Flow cytometric measurement of EdU incorporation in mouse tumors (n = 6). **g,** Flow cytometric analysis of HER2 expression in mouse tumors (n = 6). **h,** Experimental schematic of the HER2-overexpressing MFC gastric cancer xenograft model. **i,** Representative images of dissected tumors from the MFC model. **j,** Tumor growth curves in different treatment groups. **k,** Final tumor volume in the MFC model (n = 6). **l,** Final tumor weight in the MFC model (n = 6). **m,** Body weight monitoring in different treatment groups. **n-o,** Representative IHC staining images (scale bars, 50 μm) and quantification of HER2 expression in the PDX model (n = 6, four random fields per tissue section were analyzed). **p,** Body weight monitoring in HER2-low expression model. **q-t,** Serum biochemical analyses of ALT (q), AST (r), BUN (s), and Cr (t) in mouse treated with K1171-pe (n = 6). ALT, alanine aminotransferase; AST, aspartate aminotransferase; BUN, blood urea nitrogen; Cr: creatinine. *p*-values were determined by Student’s *t*-test (a, l, and q–t) or Mann-Whitney *U* test (d, f, g, k, and o); ns, not significant. Data are presented as mean ± SD.

**Figure. S7 HER2 lactylation and its therapeutic implications in cancer. a,** Western blot analysis of HER2 expression in lymphocytes. **b,** Real-time qPCR analysis of lymphocyte effector function markers (n = 3). **c,** Western blot analysis of HER2 expression in fibroblasts. **d,** Real-time qPCR analysis of fibroblast activation markers (n = 3). **e,** Western blot analysis of HER2 expression in macrophages. **f,** Real-time qPCR analysis of macrophage M1 markers and M2 markers (n = 3). **g,** Western blot analysis of AARS1 expression in trastuzumab-sensitive and trastuzumab-resistant gastric cancer patient tissues (n = 6 per group). **h,** Quantification of AARS1 expression in gastric cancer tissues across American Joint Committee on Cancer (AJCC) stages I–IV (n = 96). *p*-values were determined by Student’s *t*-test (b, d, f, and g) or Mann-Whitney *U* test (h); ns, not significant. Data are presented as mean ± SD.
